# Liquid flows induced in a rotating drum with different fill ratios

**DOI:** 10.1038/s41598-024-84579-7

**Published:** 2025-01-14

**Authors:** Daeun Lee, Jaebeen Lee, Seok Min Choi, Sangtak Lee, Hyungmin Park

**Affiliations:** 1 Department of Mechanical Engineering, Seoul National University, Seoul, 08826 Korea; 2 Samsung Research, Samsung Electronics Co., Ltd, Seoul, 06765 Korea; 3 Institute of Advanced Machines and Design, Seoul National University, Seoul, 08826 Korea

**Keywords:** Rotating drum, Fill ratio, Flow structure, Free surface, Particle image velocimetry, Mechanical engineering, Chemical engineering

## Abstract

In the present study, we experimentally investigate the liquid flow induced in a rotating drum (cylindrical tank with a short aspect ratio) aligned horizontally, focusing on the variation in the time-averaged and fluctuating flow structures with different fill ratios. For each fill ratio, controlled by varying the water height, we measure the velocity fields at different cross-sectional planes with particle image velocimetry while varying the rotational speed of the drum. Compared to the condition of a fill ratio of 1.0, in which the liquid inside the drum rotates forming a large-scale (solid-body rotation) organized flow structure, a substantial asymmetric flow structure shows up in partially-filled conditions driven by the imbalance between (i) the momentum diffusion along the radial direction and the centrifugal acceleration, and (ii) the downward (gravitational) flux of the induced flow. In addition to the mean flow structure, we examine the fluctuating velocity fields together with the dynamics of the free surface, and we also briefly discuss the difference between the liquid flow and granular (particle) flow in a partially-filled drum. We think that the present results provide valuable insights on the partially-filled liquid drum toward various engineering applications.

## Introduction

The rotating drum (circular cylindrical chamber with a short aspect ratio) aligned horizontally, whether it is fully or partially filled with fluid (mostly liquid) and/or solid particles, is a geometry encountered in many engineering applications for mixing, drying, coating, and cleaning^1–4^. For the particulate granular bed motion in a partially-filled rotating drum, the different flow regimes have been well established in terms of the Froude number, i.e., rotational speed, which is closely associated with the transport phenomena occurring inside the drum^5–7^. For example, six particle-flow regimes have been classified depending on the rotational speed in the range of 0.1–300 rpm (corresponding Froude number *Fr* = 5.6 × 10^− 7^ – 0.5 × 10^1^)^8,9^. Here, *Fr* is defined as $\:{\omega\:}^{2}d/g,$ where $\:{\upomega\:}$ (= *N* × 2$\:{\uppi\:}$/60) is the rotational speed of the drum. At very low rotational speeds (~ 1 rpm), the particles at the bed bottom are lifted and fall back down along the interface on top, causing a slumping motion. As the rotational speed increases (~ 5 rpm), the time interval between successive avalanches becomes shorter, and the flow experiences a transition period because of the competition between gravity and centrifugal forces, resulting in unstable particle beds. At higher rotational speeds (~ 40 rpm), the periodic slumping of the bed transitions to the rolling regime, characterized by the continuous movements of particle-layer over the bed surface. In this regime, the flow exhibits a flat interfacial morphology, with a thin layer of fast-moving particles on top and the majority of particles moving slowly at the bottom. With a rotational speed as fast as ~ 150 rpm, the solid particles in the upper corner (or apex) of the bed ride higher up the wall before being detached, resulting in an S-curved surface with a clear shoulder and tail, known as the cascading regime. The particles gain the energy that is sufficiently strong at the rotational speeds of ~ 200 rpm so that they are thrown off the bed surface into the space beyond the midpoint (cataracting regime). Beyond this speed (*Fr* is much larger than 1.0), the particles move along the inner edge of the cylinder. This centrifugal motion is characterized by the attachment of particles to the drum wall as a uniform layer of solid particles with little relative motion between the particles.

Compared to the particulate flows induced in a rotating drum, some previous studies have focused on the analysis of liquid-phase flow patterns (liquid-film to be specific) formed on the rotating drum wall at high rotational speeds (600–1200 rpm) and the boundary-layer development in a rimming flow^10,11^. Notably, Ivanova et al.^12^ observed the collapse of the centrifuged layer along the inner wall and the generation of inertial waves at the surface when the rotational speed exceeds 420 rpm with varying the fill ratio of distilled water between 0.05 and 0.9. In contrast, Olitskii et al.^13^ investigated the conditions under which a reverse flow, opposing the direction of wall rotation, occurs by varying the fill ratio from 0.2 to 0.58 with a relatively higher-viscosity (11 cP) fluid. Thoroddsen and Mahadevan^14^ analyzed the sloshing instabilities developing on the fluid surface along the wall and the formation of a shark-teeth pattern in the axial direction in liquids with viscosities ranging from 20 to 1020 cP, when the centrifugal force (rotational speeds up to 480 rpm) become significantly stronger than the gravitational force. However, it still requires more investigation on how the liquid flow is generated at lower rotational speeds when the rimming does not occur.

When the rotating drum is fully filled with a liquid, as the simplest condition, the entire internal flow field inside the drum is driven by the shear stress between the fluid and the drum wall due to viscosity. As a result, the momentum diffusion from the rotating wall towards the center of rotation governs the flow in the fully developed regime^15^. On the other hand, the problem becomes more complicated for the partially filled configurations (with liquid as a working fluid) because of the increasing contribution by the gravitational acceleration (presence of a free surface). Previously, Haji-Sheikh et al.^16^ described the free-surface shape for a ratio of the Reynolds to Froude numbers within the range of 100–5000 and visualized the corresponding streamlines. Here, the Reynolds number is defined as $\:{Re}_{\omega\:}$= $\:\omega\:{d}^{2}/\nu\:$, where $\:{\upnu\:}$ is the kinematic viscosity. Similarly, Böhme et al.^17^ experimentally investigated the relative contributions of gravitational and viscous effects on the liquid within a rotating drum, as well as the impact of fill ratio on the deformation of the free surface. They suggest that the velocity field within the liquid needs to be measured and analyzed as future research. Dyakova and Polezhaev^18^ revealed that the azimuthal steady streaming velocity is proportional to the square of the ratio of gravitational to centrifugal forces by varying the fill ratio from 0.1 to 0.6. However, there is a lack of comprehensive data for the liquid-phase velocity fields inside partially-filled rotating drums. In addition, a comparative analysis between the particle- and liquid-phase flows in a rotating drum is necessary to understand the underlying mechanism clearly.

Therefore, in the present study, we experimentally investigate the liquid (water) flows induced in fully and partially filled rotating drums aligned horizontally, focusing on how the presence of a free surface affects the mean and fluctuating flow fields. Using planar particle image velocimetry, we measure the flow structures on different cross-sectional planes while varying the fill ratio and rotational speed. Because of the high density of the filling fluid considered, the radial momentum diffusion by the centrifugal acceleration is counterbalanced by the downward inertia (gravitational effect), which becomes more complicated for the partially filled cases. To the best of our knowledge, this is the first experimental investigation to discuss the variation of the liquid flow induced in the rotating drum depending on the fill ratio, which will be useful in enhancing the performance of processing systems based on a rotating drum.

## Results and discussion

### Flows in a fully-filled rotating drum

**Fig. 1:**
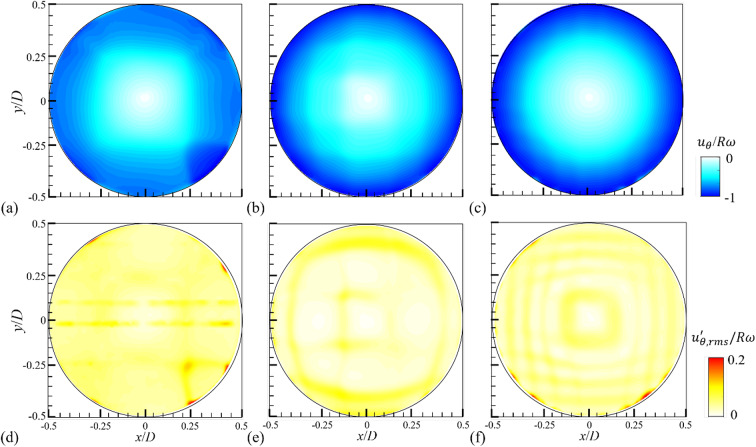
Contours of the time-averaged (**a**–**c**) and root-mean-square of fluctuating (**d**–**f**) azimuthal velocity for a fully-filled case measured at the mid-plane (z*/W* = 0): (a, d) *N* = 10 rpm; (**b**,**e**) 30 rpm; (**c**,**f**) 50 rpm.

First, we briefly discuss the liquid flows induced in the fully filled (*H/D* = 1.0) rotating drum by varying the rotational speed, measured at the mid-plane (*z/W* = 0). For all considered rotational speeds, the azimuthal velocity ($\:{u}_{\theta\:}$) fields show that the liquid flow globally rotates in the same direction as the drum, while the contour becomes closer to a circular (annular) shape with increasing centrifugal acceleration (i.e., rotational speed, *N*) (Fig. 1a-c). This suggests that the flow reaches the solid-body rotation, characterized by a linear distribution of azimuthal velocity^19,20^. Near the wall at *r*/*D* = 0.45–0.5, there exists a boundary-layer like region with a relatively high $\:|{u}_{\theta\:}/R\omega\:|$ (> 0.9) of which the thickness is quite similar for considered rotational speeds because the flow inside the drum eventually reaches a fully-developed state. The root-mean-square of the fluctuating azimuthal velocity ($\:{u}_{\theta\:,rms}^{{\prime\:}}$), on the other hand, shows a homogeneous distribution inside the drum with a lower value (Fig. 1d–f). Compared to the azimuthal velocity, in contrast, the magnitude of the radial velocity ($\:{u}_{r}$) is quite small (less than 10% of $\:{u}_{\theta\:}$), which decreases more as the drum rotates faster (see Fig. S1 in the supplementary materials). Additionally, we observed that there exists no strong axial flow (along the *z*-direction) in the fully-filled condition, which can be inferred from the fact that the time-averaged and fluctuating velocity fields measured near the wall (*z*/*W* = 0.25) (Fig. S2 in the supplementary materials) are quite similar to those at the mid-plane (*z*/*W* = 0).

**Fig. 2:**
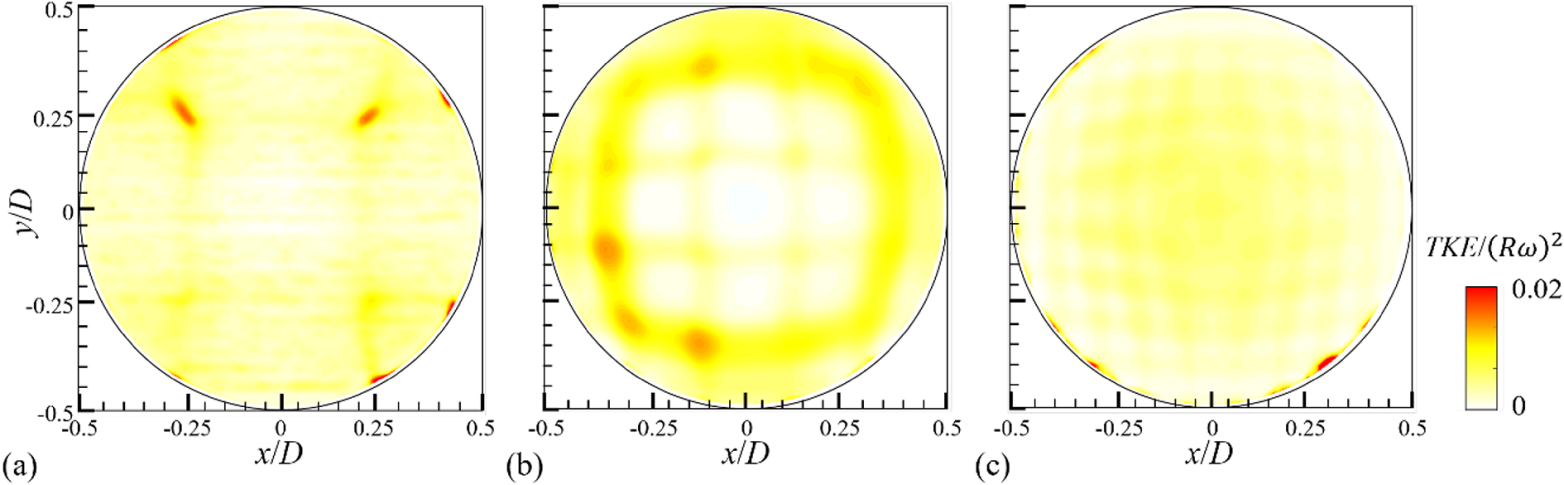
Contours of the turbulent kinetic energy (*TKE* = $\:0.5({{u}^{{\prime\:}}}_{r}^{2}+{{u}^{{\prime\:}}}_{\theta\:}^{2})$) for the fully-filled condition with different rotation speed (*N*) measured at the mid-plane (z*/W* = 0): (**a**) *N* = 10 rpm; (**b**) 30 rpm; (**c**) 50 rpm.

In Fig. 2, the turbulent kinetic energy (*TKE* = $\:0.5({u}_{r}^{{\prime\:}2}+{u}_{\theta\:}^{{\prime\:}2})$) is compared as an indirect index for the mixing performance of the rotating drum, considering possible engineering applications (e.g., washing machine, mixing chamber, reactor, and so on). Since the flow is induced primarily along the azimuthal direction, the trend of *TKE* distribution depending on varying *N* is similar to that of $\:{u}_{\theta\:,rms}^{{\prime\:}}$, and the energy level does not vary significantly with *N* and has a uniform distribution. When the drum is fully filled with a liquid, despite the low aspect ratio of the drum, similar flow structures are measured along the axial (*z*) direction, except for the positions very near the walls (*z/W* = ± 0.5). This indicates that, in the absence of a free surface, the flow is dominated by rotational inertia and gravity. As we will discuss below, there exists a case (small fill ratio rotating slowly) for partially filled conditions where the axial flow is induced.

### Time-averaged flows in a partially-filled rotating drum

**Fig. 3:**
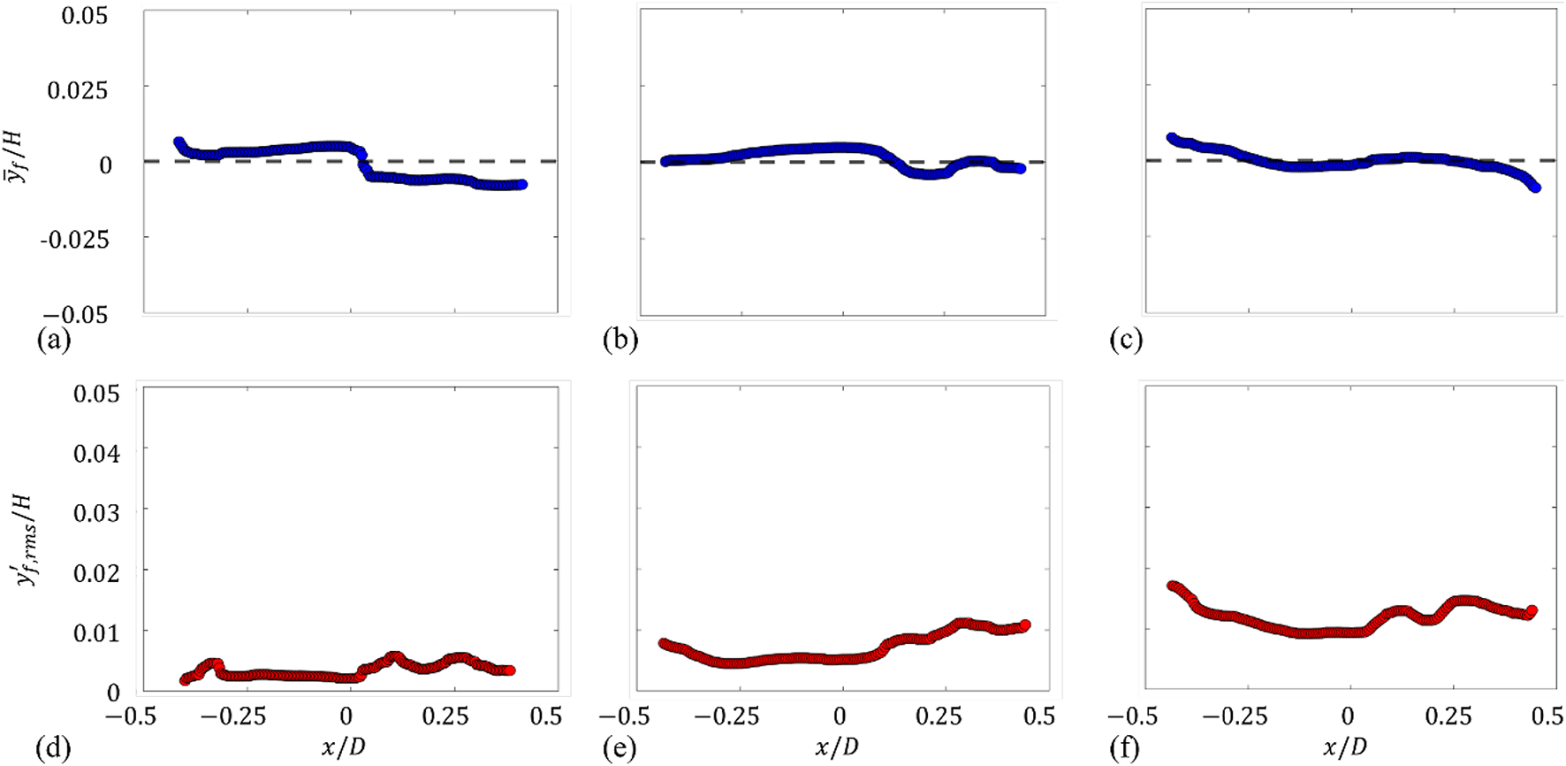
Variation of the averaged (**a**–**c**) and root-mean-squared fluctuating (**d**–**f**) water height (*y*_f_ measured from the stationary case) of partially-filled (*H/D* = 0.5) condition: (**a**,**d**) *N* = 10 rpm; (**b**,**e**) 30 rpm; (**c**,**f**) 50 rpm.

Before we discuss the flow induced in a partially-filled rotating drum, the possible influence of the drum rotation on the free surface is examined (please see Methods section for the details of measurement technique). Figure 3 shows the change in the averaged ($\:{\stackrel{-}{y}}_{f}$) and root-mean-square of fluctuating ($\:{y}_{f,rms}^{{\prime\:}}$) height of free surface measured from the stationary (i.e., without rotation) position. Shown in the case is the water height of *H*/*D* = 0.5 (fill ratio of 0.5). When the rotational speed is relatively low, the variation of the mean free-surface height is less than 1% of the water height (*H*) and the horizontal profile is maintained to be quite flat (Fig. 3a). At 50 rpm, the mild slope of the profile appears near both walls (*x/D* = $\:\pm\:$0.5), but its amplitude (less than 2% of *H*) is still too small to affect the global flow (Fig. 3 c). Thus, it can be understood that the free surface maintains a horizontal orientation during the rotation, except at the position very near the drum wall. Since the normal direction to the free surface is aligned with the direction of acceleration, this indicates that the centrifugal acceleration ($\:{a}_{c}={\omega\:}^{2}d$; see Methods for the definitions of the variables) by the rotating drum is not strong enough to alter the direction of local gravitational acceleration effectively (note that the Froude number, the ratio of centrifugal acceleration to gravitational accelerations is quite smaller than 1.0; see Methods). According to Phillips^21^, the pressure gradients driven by the gravitational and centrifugal accelerations are oppositely signed for the partially-filled cases and a necessary condition for the stability of the flow is the positive net radial pressure gradient. In the present experimental conditions, the rotational speed is not sufficiently fast and the Froude number (ratio of centrifugal to gravitational accelerations) is much smaller than 1.0 (see Methods). This implies that gravitational forces have a relatively strong effect on maintaining a flat free surface compared to rimming flow^10,22^. Furthermore, Romanò et al.^23^ explained that the flat air-water interface is attributed to the dominant effect of gravitational force over centrifugal and capillary forces, which is characterized by very small Froude number ($\:Fr\ll\:1.0$). On the other hand, the root-mean-square of fluctuating height ($\:{y}_{f,rms}^{{\prime\:}}$) of free surface is quite small (less than 2.0% of *H*) in all cases (Fig. 3d-f). The fluctuation also increases with the rotational speed, but its amplitude is again marginally small.

**Fig. 4:**
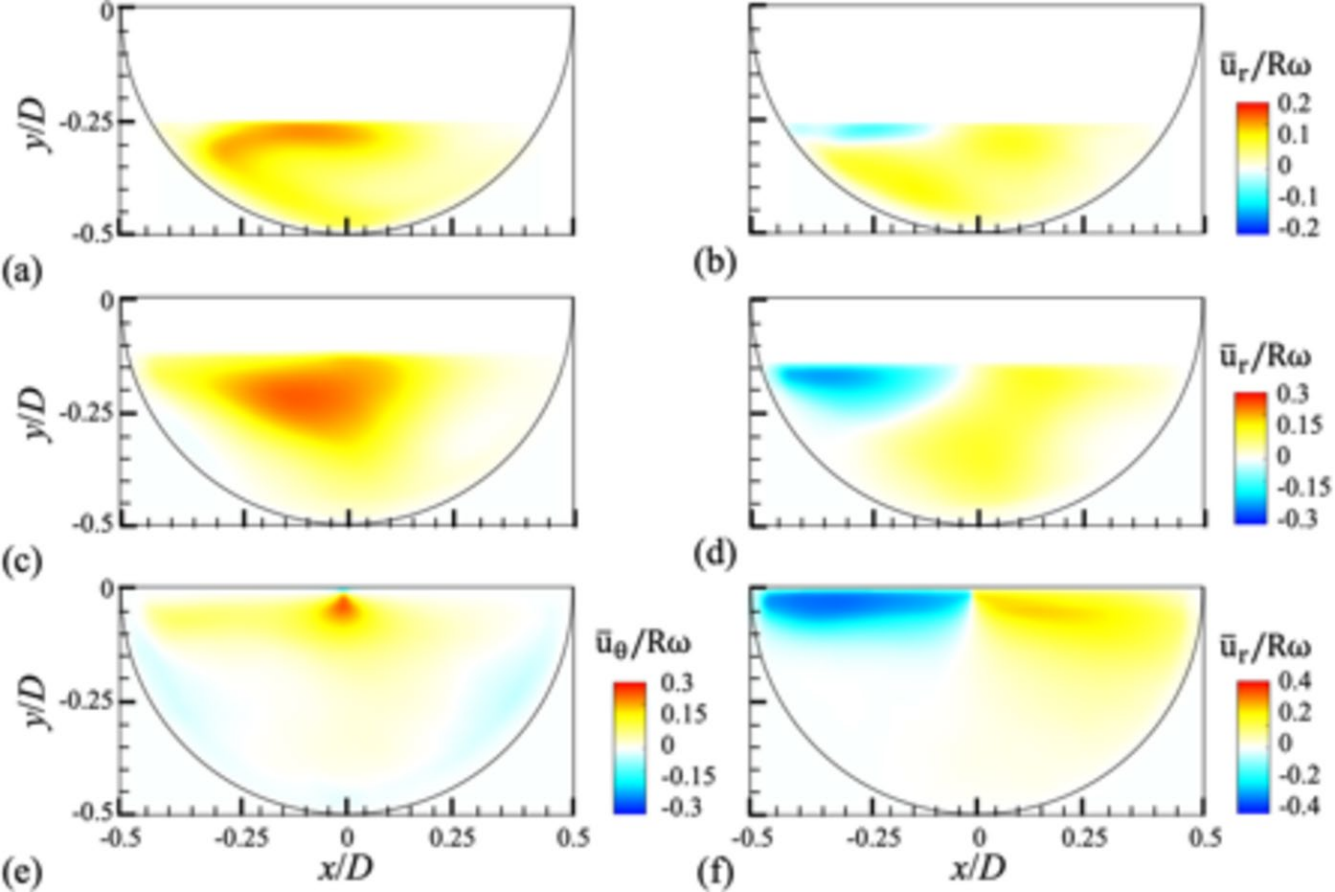
Contours of time-averaged azimuthal (**a**,**c**,**e**) and radial (**b**,**d**,**f**) velocities for different water height (*H*) at the mid plane (*z/W* = 0): (**a**,**b**) *H/D* = 0.25; (**c**,**d**) 0.375; (**e**,**f**) 0.5. The rotational speed of the drum is *N* = 30 rpm.

Figure 4 shows the contours of the azimuthal and radial velocities for the liquid flow induced in the partially filled drum at *N* = 30 rpm (see Figs. S3 and S4 in the supplementary materials for all rotational speeds considered). Note that similar flow structures are roughly retained along the axial direction even for the partially filled conditions, so we focus on the flows measured at the mid-plane (*z/W* = 0). When the fill ratio is *α* ≤ 0.5, the positive (counter-clockwise) components of azimuthal velocity is dominant, especially near the middle of the free surface (Fig. 4a, c, e), indicating that the liquid flow is not synchronized with the direction of the drum rotation like the fully-filled condition (Fig. 1). With increasing water height (*H*), i.e., the fill ratio, the layer of negative (clockwise) azimuthal velocity near the wall becomes thicker (Fig. 4e). Due to the presence of the free surface, the magnitude of the radial velocity is relatively larger near it compared to the bulk region (Fig. 4b, d, f), which becomes clearer with increasing fill ratio (Fig. 4f). When the fill ratio is small (Fig. 4b), the radial velocity is mostly positive except on the left side of the free surface. While the magnitude of the radial velocity is much smaller than the azimuthal component in a fully filled condition, they are comparable to each other for partially filled cases, indicating that the influence of centrifugal acceleration and viscous stress is not dominant over gravitational force.

**Fig. 5:**
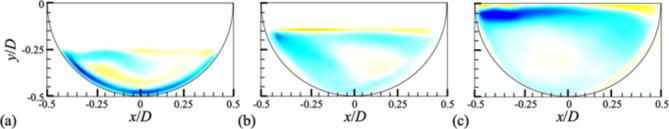
Contours of axial vorticity for different water height (*H*) at the mid plane (*z/W* = 0): (**a**) *H/D* = 0.25; (**b**) 0.375; (**c**) 0.5. The rotational speed of the drum is *N* = 50 rpm.

**Fig. 6:**
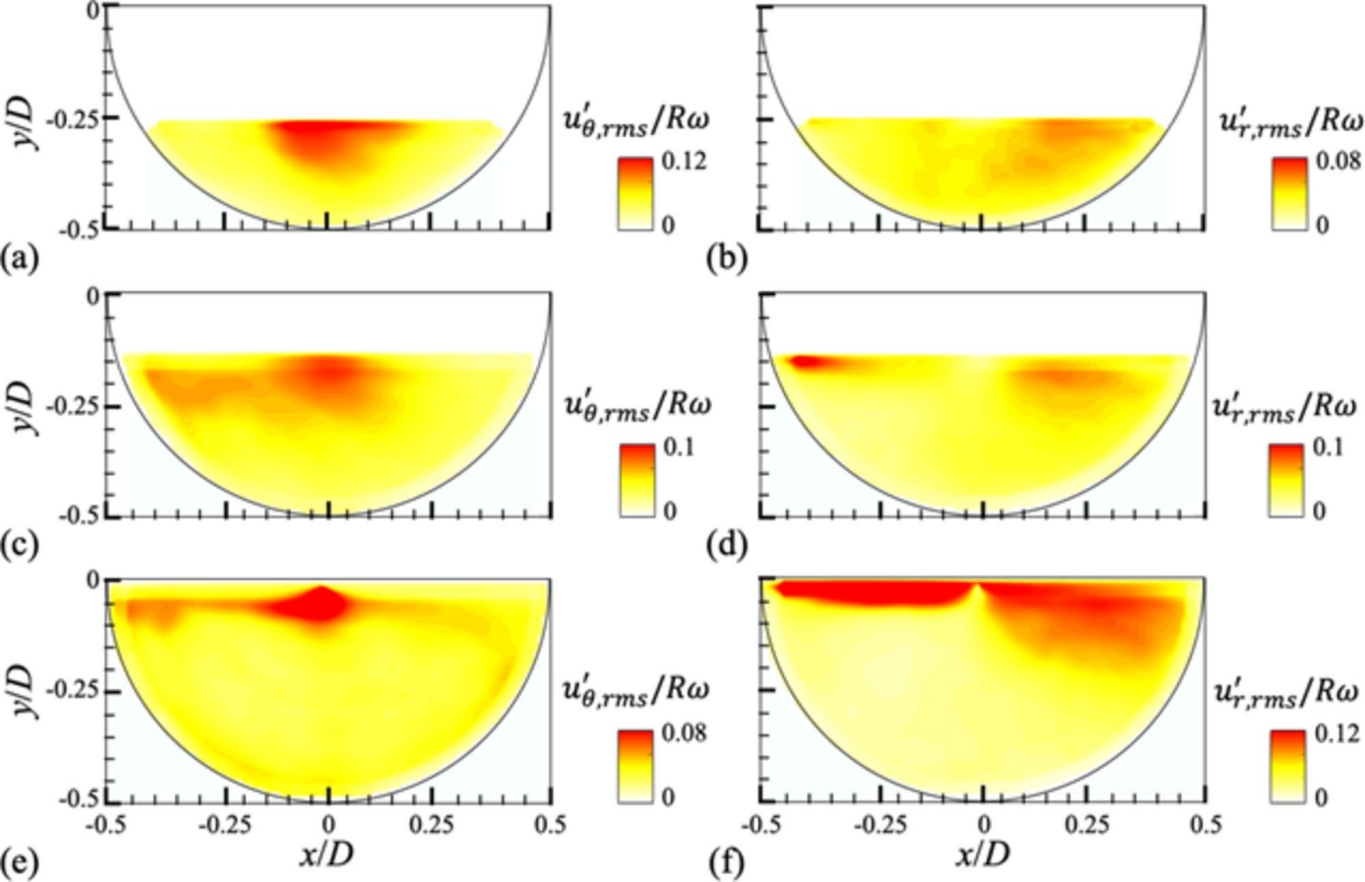
Contours of the root-mean-square fluctuating azimuthal ($\:{\varvec{u}}_{\varvec{\theta\:}}^{\varvec{{\prime\:}}})$ (**a**,**c**,**e**) and radial ($\:{\varvec{u}}_{\varvec{r}}^{\varvec{{\prime\:}}}$) (**b**,**d**,**f**) velocities for different water height (*H*) at the mid-plane (*z/W* = 0): (**a**,**b**) *H/D* = 0.25; (**c**,**d**) 0.375; (**e**,**f**) 0.5. The rotational speed of the drum is *N* = 10 rpm.

Figure 5 shows the contours of time-averaged axial vorticity ($\:{\stackrel{-}{\omega\:}}_{z}$), normalized by the rotational speed of the drum, for different *H* while the rotational speed is fixed at *N* = 50 rpm. For all rotational speeds, overall, the same vortical structures are formed for a given fill ratio (see Fig. S5 in the supplementary materials). As shown, the vortical structure in the drum is not axisymmetric about the rotational axis as in the fully filled cases; rather, the region of strong shear appears locally depending on the fill ratio (water height). At low water heights, the vorticity magnitude has a sharp peak near the rotating wall (Fig. 5a), but it transitions to a broader peak right below the free surface as *H* increases (Fig. 5b,c). This also supports that the contribution of viscous diffusion and centrifugal acceleration may affect the flow at a small fill ratio, but the effect of the free surface (fast attenuation of the wall-induced flow) associated with gravitational force becomes stronger as *H* increases.

### Fluctuating velocity fields in a partially-filled rotating drum

The agitated (fluctuating) flow structures are plotted in terms of the contours of the root-mean-square of the fluctuating azimuthal ($\:{u}_{\theta, rms\:}^{{\prime\:}})$ and radial ($\:{u}_{r, rms}^{{\prime\:}})$ velocities (Fig. 6). Shown in the figure is the case of *N* = 10 rpm, and the contours for all rotational speeds considered are provided in Figs. S6 and S7 in the supplementary materials. In overall, it is found that the level of fluctuating velocity in the partially-filled cases is higher compared to the fully-filled condition (Fig. 1), which is attributed to the presence of free surface. This is supported by the fact that both $\:{u}_{\theta\:,rms}^{{\prime\:}}$ and $\:{u}_{r,rms}^{{\prime\:}}$ have a peaking region near the free surface, of which the non-dimensionalized magnitudes tend to decrease with increasing the rotational speed (Figs. S6 and S7). As the centrifugal acceleration increases, the unsteady velocity fluctuation near the free surface is relatively attenuated. While the levels of $\:{u}_{\theta\:,rms}^{{\prime\:}}$ and $\:{u}_{r,rms}^{{\prime\:}}$ are comparable to each other, their variations with water height show different trends: $\:{u}_{\theta\:,rms}^{{\prime\:}}$ ($\:{u}_{r,rms}^{{\prime\:}}$) decreases (increases) with increasing *H*. Since the velocity fluctuations are concentrated near the free surface, the increase of centrifugal force by the larger water volume would result in the reduction of $\:{u}_{\theta\:,rms}^{{\prime\:}}$; however, along the radial direction, $\:{u}_{r,rms}^{{\prime\:}}$ on the free surface is enhanced following the strong radial flows (see Fig. 4b,d,f).

**Fig. 7:**
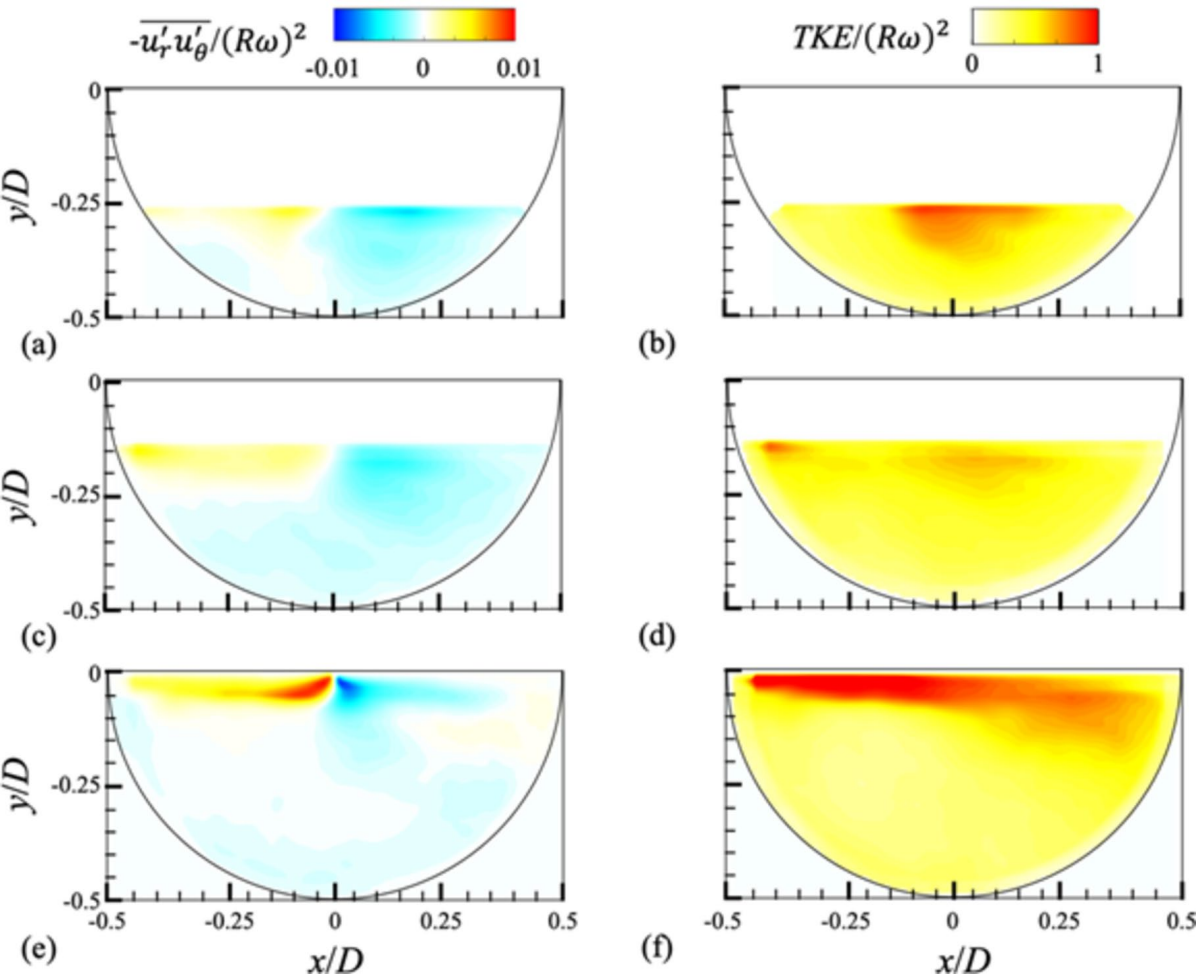
Contours of the Reynolds stress (**a**,**c**,**e**) and turbulent kinetic energy (*TKE* = $\:0.5({u}_{r}^{{\prime\:}2}+{u}_{\theta\:}^{{\prime\:}2})$) for different water height (*H*) at the mid plane (z*/W* = 0): (**a**,**b**) *H/D* = 0.25; (**c**,**d**) 0.375; (**e**,**f**) 0.5. The rotational speed of the drum is *N* = 10 rpm.

For the cases (*N* = 10 rpm) shown in Fig. 6, the corresponding Reynolds stress ($\:-\stackrel{-}{{u}_{r}^{{\prime\:}}{u}_{\theta\:}^{{\prime\:}}}$) and turbulent kinetic energy (TKE = $\:0.5({u}_{r}^{{\prime\:}2}+{u}_{\theta\:}^{{\prime\:}2})$) with *H* are shown in (Fig. 7). The cases of *N* = 30 and 50 rpm are additionally provided in Figs. S8 and S9 in the supplementary materials. Compared to the case of fully-filled condition (Fig. 2), both indices have increased globally like the fluctuating velocity components. Following the distributions of $\:{u}_{\theta\:,rms}^{{\prime\:}}$ and $\:{u}_{r,rms}^{{\prime\:}}$, the larger Reynolds stress and turbulent kinetic energy are generated near-free surface region, which are enhanced more with increasing *H*. As the rotational speed increases, the Reynolds stress distributed near the free surface and the turbulent kinetic energy normalized by $\:{\left(R\omega\:\right)}^{2}$ tend to decrease (Figs. S8 and S9). This indicates that the influence of free surface is reduced as the contribution of centrifugal acceleration increases, supporting our discussion so far.

### Further discussions

So far, the liquid flows induced in the fully- and partially-filled rotating drums have been discussed based on the flow fields measured on the mid-plane (*z*/*W* = 0) because of the relatively stronger contribution of centrifugal and gravitational forces over the viscous force (i.e., wall effect) despite the low aspect ratio of the drum. For the present configuration, the relevant forces (per unit volume) to affect the flows are the centrifugal ($\:{f}_{c}=\rho\:{\omega\:}^{2}d),$ gravitational ($\:{f}_{g}=\rho\:g)$, and viscous ($\:{f}_{v}=\nabla\:\cdot\:\tau\:$) forces, where $\:\rho\:$ is liquid density and $\:\tau\:$ is the viscous stress. Considering that the viscous stress is scaled as $\:\tau\:=\mu\:(\partial\:{u}_{\theta\:}/\partial\:r)\sim\mu\:(\omega\:H/H)\sim\mu\:\omega\:$, the order of magnitudes of the force ratios are estimated as $\:{f}_{v}/{f}_{c}$ = ***O***(10^−2^) and $\:{f}_{v}/{f}_{g}$ = ***O***(10^−4^), respectively, indicating the substantially weaker contribution by the viscous momentum diffusion. This is consistent with our observation such that the global flow structures demonstrated by the contours of averaged and fluctuating velocities are maintained to be the similar along the axial direction. However, there is a case in which the dependency on the water height and rotational speed shows up differently because of the wall (i.e., *z*/*W* = ± 0.5) effect.

**Fig. 8:**
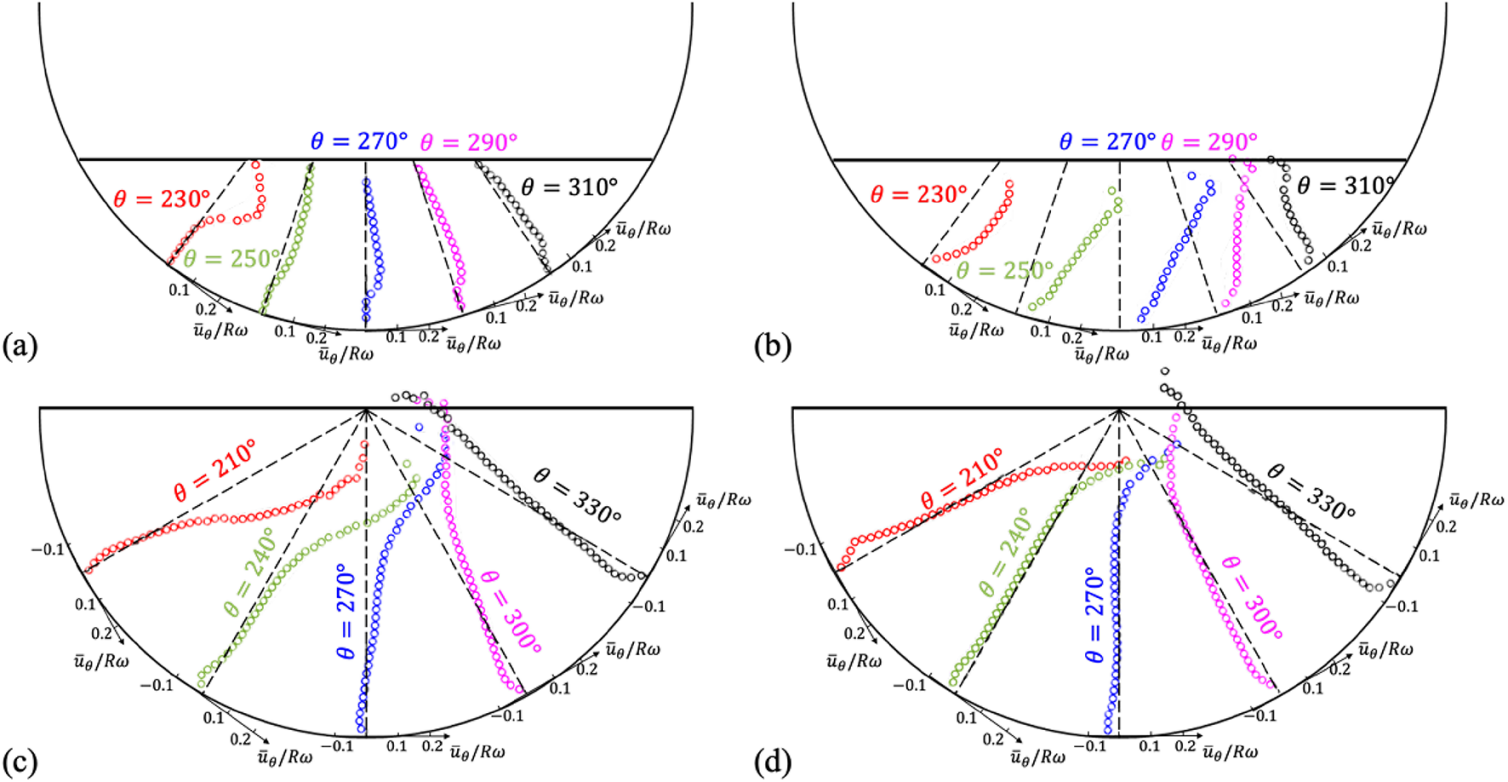
Radial profiles of the time-averaged azimuthal velocity, $\:{\stackrel{-}{\varvec{u}}}_{\varvec{\theta\:}}\left(\varvec{r}\right)$, at specific angles ($\:\varvec{\theta\:}$), measured at *z*/*W* = 0.25 (**a**,**c**) and 0 (b, d): (**a**,**b**) *H/D* = 0.25 and *N* = 10 rpm; (**c**,**d**) *H*/*D* = 0.5 and *N* = 50 rpm.

**Fig. 9:**
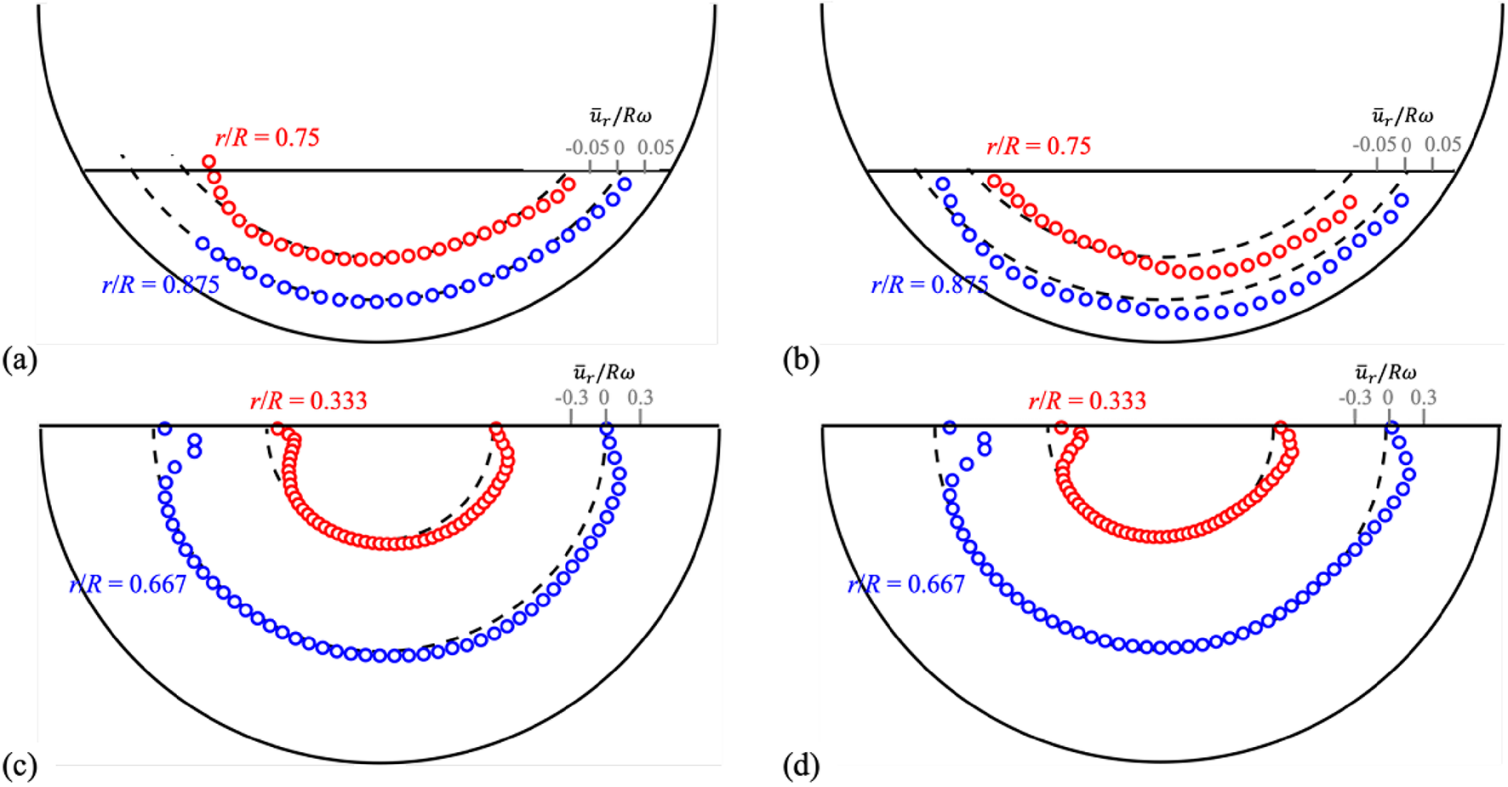
Azimuthal profiles of the time-averaged radial velocity, $\:{\stackrel{-}{\varvec{u}}}_{\varvec{r}}\left(\varvec{\theta\:}\right)$, at specific radial positions ($\:\varvec{r}$), measured at *z*/*W* = 0.25 (**a**,**c**) and 0 (**b**,**d**): (**a**,**b**) *H*/*D* = 0.25 and *N* = 10 rpm; (**c**,**d**) *H*/*D* = 0.5 and *N* = 50 rpm.

**Fig. 10:**
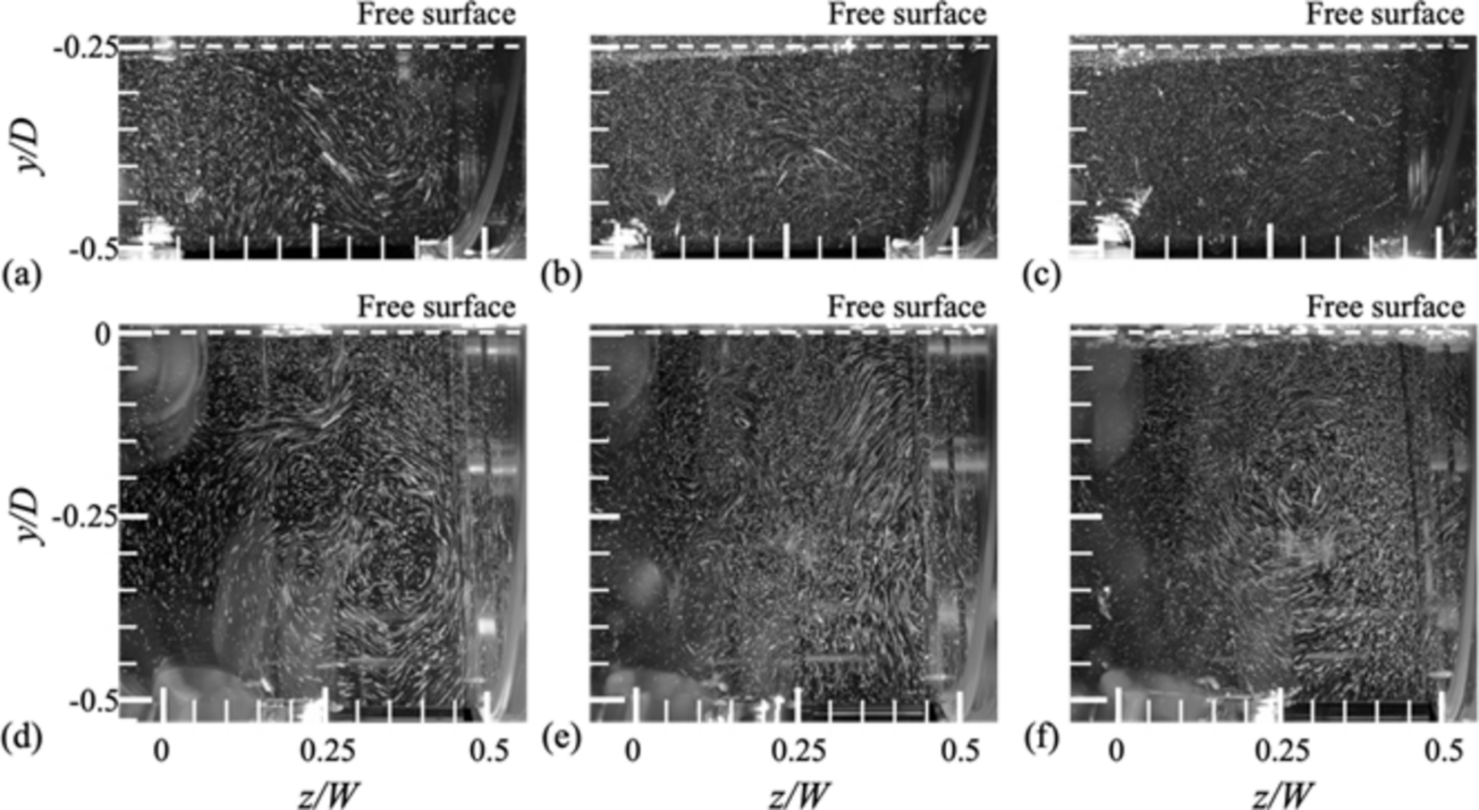
Visualization of the flows at side (*y*-*z*) plane for *H*/*D* = 0.25 (**a**–**c**) and 0.5 (**d**–**f**): (**a**,**d**) *N* = 10 rpm; (**b**,**e**) 30 rpm; (**c**,**f**) 50 rpm.

**Fig. 11:**
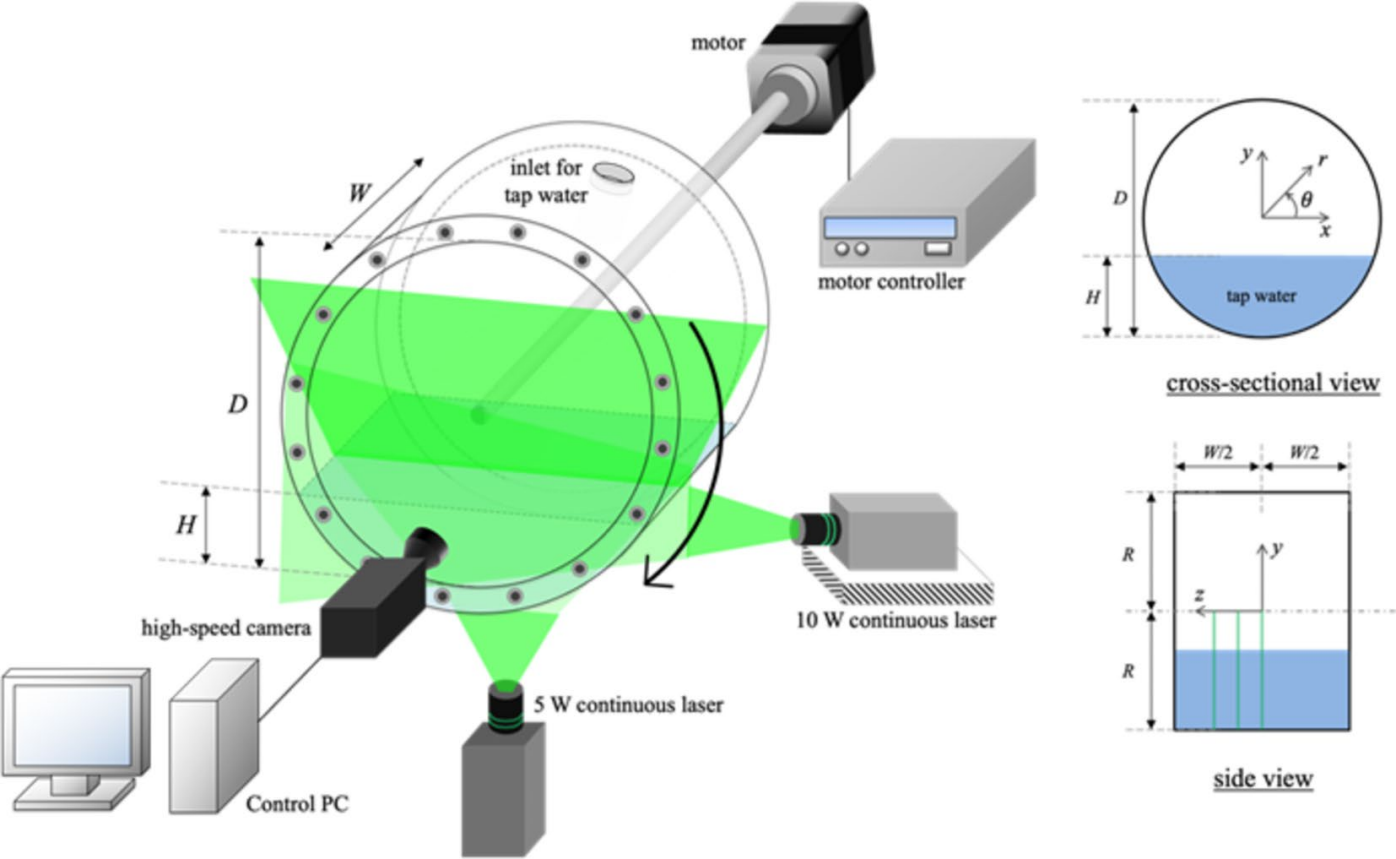
Schematic diagram for the experimental setup of the rotating drum facility with the particle image velocimetry (PIV) to measure the velocity fields at three x-y planes (*z*/*W* = 0, 0.125, and 0.25; highlighted with green lines in side view).

In Figs. 8 and 9, we have compared the azimuthal and radial velocity profiles at specific positions on the planes of *z*/*W* = 0 (mid-plane) and *z*/*W* = 0.25 (closer to the drum wall) for two cases (see Figs. S10 and S11 in the supplementary materials for the same velocity profiles in Cartesian coordinates): one is the case of *H*/*D* = 0.25 and *N* = 10 rpm, and the other is the case of *H*/*D* = 0.5 and *N* = 50 rpm. Indeed, except the first case, the effects of the drum wall on the flow inside are not significant. With the azimuthal velocity profiles $\:{\stackrel{-}{u}}_{\theta\:}\left(r\right)$, the flow induced by the interaction between the centrifugal and gravitational forces are more clearly understood; that is, at all axial (*z*) positions, the negative (clockwise) $\:{\stackrel{-}{u}}_{\theta\:}$ is induced very near the drum wall following the rotational direction but the positive (counter-clockwise) $\:{\stackrel{-}{u}}_{\theta\:}$ is formed near the free surface (Fig. 8). At a fixed radial position, the radial velocity $\:{\stackrel{-}{u}}_{r}\left(\theta\:\right)$ is almost negligible except near-free surface region, at which the surface flow is directed toward the righthand side following the direction of drum rotation (Fig. 9). When the water height and rotational speed are low, both the radial and azimuthal velocities are decelerated much near the wall (*z*/*W* = 0.25) compared to the mid-plane (*z*/*W* = 0) (Figs. 8a and 9a). However, for the higher water height and rotational speed, the wall effect is confined to very near the wall and the similar velocity profiles are maintained along the axial direction (Figs. 8c and 9c).

To further understand the flow structures on the side plane, we obtained the corresponding pathlines by accumulating the motion (images) of particles (the same particles used for particle image velocimetry; see Methods) for 0.3–0.5 s^24,25^. Due to the curvature of the cylindrical drum and obstructions by the appendages, it was not possible to measure the quantitative velocity distribution in detail. Figure 10 shows the representative instantaneous flow fields for the water height of *H/D* = 0.25 and 0.5 while varying the rotational speed. For lower rotational speeds, large-scale flows in the axial direction are observed (Fig. 10a, d), which tend to be mitigated as *N* increases (Fig. 10c, f). While this kind of axial flow is not observed for the fully filled condition, its influence is not strong enough to alter the velocity distributions on the x-y planes substantially (Figs. 8 and 9).

As we have discussed, the liquid flow in a partially-filled rotating drum is strongly governed by the gravitational force because of the relatively large density of the liquid, and thus the gas-liquid interface is not inclined along the rotational direction like the granular beds in a rotating chamber^26^. Considering the above-mentioned relevant forces, it is derived that the summation of centrifugal ($\:{f}_{c}=\rho\:{\omega\:}^{2}d$) and viscous ($\:{f}_{v}=\nabla\:\bullet\:\tau\:$) forces needs to exceed the gravitational ($\:{f}_{g}=\rho\:g)$ force, in order to have a morphologically similar flow regime to the particulate beds. Since the hydraulic diameter (*d*) is proportional to water height (*H*), we can draw the scaling relation to satisfy $\:{f}_{c}+{f}_{v}>{f}_{g}$ as $\:\rho\:{\omega\:}^{2}H+\mu\:\omega\:/H>\rho\:g$. For the centrifugal acceleration of $\:{a}_{c}={\omega\:}^{2}d\sim{\omega\:}^{2}H$, it is further developed into $\:g/{a}_{c}<1+\mu\:/\rho\:\omega\:{H}^{2}$ or $\:g/{a}_{c}<1+1/{Re}_{\omega\:}$ (note that the Reynolds number is $\:{Re}_{\omega\:}=\rho\:\omega\:{d}^{2}/\mu\:\sim\rho\:\omega\:{H}^{2}/\mu\:$). If the centrifugal acceleration ($\:{a}_{c}$) is larger than the gravitational acceleration ($\:g/{a}_{c}<1.0)$, which corresponds to *N* > 100 rpm for the present conditions, obviously this relation will be satisfied irrespective of the Reynolds number. When the centrifugal acceleration is fixed, then the Reynolds number needs to be smaller by increasing the viscosity of the fluid, for example, which will resemble the particulate beds in a rotating drum.

### Concluding remarks

In the present study, we have experimentally investigated the liquid (water) flow induced in the fully and partially filled rotating drums aligned horizontally (i.e., with the axis parallel to the horizon), focusing on understanding the effects of the free surface on the distribution of averaged and fluctuating velocities. We varied the water height (i.e., fill ratio) and the rotational speed of the drum, targeting engineering applications involving coating, drying, and cleaning processes. It was clearly shown that the liquid flows induced in partially filled conditions are quite different from the fully filled case (solid-body rotation) where the entire flow domain is affected by viscous diffusion and centrifugal acceleration, resulting in the axisymmetric annular distribution of the velocity fields. With the existence of a free surface, the contribution of gravitational force, counteracting the dragging movement of the induced liquid near the drum wall, appears to be comparable to or dominant over centrifugal and viscous forces. As a result, the flow symmetry is broken, and the strong mean and fluctuating velocities are concentrated near the free surface. As the water height (i.e., fill ratio) decreases, the effect of viscous diffusion tends to be influential (the flow near the solid surface is disturbed to the same level as that near the free surface) when the rotational speed is relatively slow. However, in most other conditions, the vortical structures and the regions of higher turbulent kinetic energy are only formed near the free surface. Driven by these flow characteristics, the levels of Reynolds stress and turbulent kinetic energy (indicating the magnitude of the agitated flow in the drum) are globally reduced for the partially filled cases.

While the dynamics of particulate (granular) beds in the same geometry (rotating drum) have been investigated in detail, the liquid flows have received less attention so far. As explained clearly, however, the underlying mechanism (competitive interaction among centrifugal, gravitational, and viscous forces) of the induced liquid flows is quite different from that of granular beds (particle-particle interaction, static friction, and gravity), and the resulting flow structures are also distinct from each other, which is to be investigated in detail as a future work. In such a context, the present experimental results are considered to be helpful in providing insights into designing and optimizing engineering platforms for various purposes. Furthermore, beyond the present single (liquid)-phase flows, the dynamics of bubbly or particle-laden flows in a rotating drum will be an interesting topic for future research.

## Methods

### Rotating drum facility

In the present experiments, a short cylindrical tank (drum) made of an acrylic is utilized as a testbed, of which the *z*-axis is aligned horizontally (Fig. 11). The inner diameter (*D* = 2*R*) of the drum is 300 mm and the depth (*W*) is 200 mm, i.e., the aspect ratio is *W*/*D* = 0.67. The thickness of the drum wall is 10 mm. The drum is filled with a tap water (at the room temperature of 20 °C) at different heights (*H*) of *H*/*D* = 0.25, 0.375, 0.5, and 1.0 (fully filled), corresponding to the fill (volume) ratio (*α*) of 0.19, 0.34, 0.5, and 1.0, respectively. The hydraulic diameter (*d*) of the filled water, which is calculated as *d* = 4*A/P*, where *P* and *A* is the perimeter and area of the water on the cross-sectional (*x*-*y*) plane, varies as *d*/*D* = 0.31, 0.46, 0.61, and 1.0. For each fill ratio, the drum is rotated in a clockwise direction at the speed of *N* = 10, 30, and 50 rpm, which is controlled by motor connected with the backside of the drum. Based on the hydraulic diameter as a characteristic length scale, the rotational Reynolds number ($\:{Re}_{\omega\:}$= $\:\omega\:{d}^{2}/\nu\:$) and Froude number (*Fr* = $\:{\omega\:}^{2}d/g$) ranges as 9.05 × 10^3^ − 4.71 × 10^5^ and 1.04 × 10^− 2^ − 8.39 × 10^− 1^, respectively^23^. Here, $\:{\upnu\:}$ is the kinematic viscosity of water and $\:{\upomega\:}$ (= *N* × 2$\:{\uppi\:}$/60) is the rotational speed of the drum. These dimensionless parameters indicate that the rotational inertia dominates the viscous effect in the present flows, and the contributions of both the gravity and centrifugal force should be considered together, of which the relative governance would be determined by the fill ratio and rotational speed. The supporting information is provided in Fig. S12 in the supplementary materials.

**Fig. 12:**
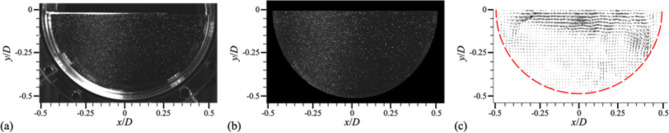
Representative examples of particle image velocimetry (PIV): (**a**) raw images obtained for PIV; (**b**) particle image after the background masked; (**c**) instantaneous velocity fields processed for the corresponding liquid flow. Here, the case of *H*/*D* = 0.5 and *N* = 10 rpm is shown.

### Particle image velocimetry (PIV)

We measured the liquid velocity fields induced by the rotating drum using a planar particle image velocimetry (Fig. 11). At different axial positions of *z*/*W* = 0, 0.125, and 0.25, the temporal evolution of the flow fields on the cross-sectional (*x*-*y*) planes were obtained at the speed of 400 fps (frames per second) using the high-speed camera (pixel size of 1280 × 800) (SpeedSense VEO E, Dantec Dynamics) equipped with a green optical filter (cut-off wavelength of 532 nm). As a tracer particle, we used hollow glass spheres (HGS-10, Dantec Dynamics) with a nominal diameter of 10 μm, which were illuminated by two green-colored (wavelength of 532 nm) continuous-wave (CW) lasers (10-W DPSS Laser, RayPower 5000, Dantec Dynamics). Since the present circular test section may cause a distortion in the data causing the non-uniform distribution of the light intensity, we located additional laser below the test section, by which it was possible to illuminate the entire cross-section (Fig. 12). It was also checked that two laser sheets form a plane at the same depth of focus. We confirmed that the liquid inside the drum reaches a fully-developed flow approximately 5 min after rotation begins by monitoring the flow field development over time (see Fig. S13 in the supplementary materials). To ensure a steady state, we obtained the instantaneous velocity field 10 min after the drum started rotating. The conventional cross correlation based on Fourier transform algorithm^27^ was applied to the pairs of consecutive particle images to evaluate the velocity vectors with a 32 × 32 pixel interrogation window (75% overlap). The erroneous vectors were detected by the normalized median test^28^ and replaced by the mean value of the neighboring vectors in a 3 × 3 grid, in terms of the instantaneous velocity field. For each condition, more than 48,000 instantaneous flow fields (see Fig. 12c for an example) were obtained and averaged to obtain the statistically converged values. The velocity fields for fully- and partially-filled rotating drums were measured in the field of view of $\:-0.5D\le\:x\le\:0.5D$, $\:-0.5D\le\:y\le\:\:0.5D$ and $\:-0.5D\le\:x\le\:0.5D$, $\:-0.5D\le\:y\le\:\:0$, respectively with a spatial resolution of 960 $\:\times\:$ 960 pixels. It was determined to cover the entire flow field for each case with sufficient accuracy. Owing to the significant distortion of the particle images caused by large curvature and rotation of the drum, with the current setup, it was not allowed to perform the quantitative measurements of the velocity fields on the side (*y*-*z*) planes. Instead, we tried to qualitatively visualize the flow patterns to help understanding the global flow structures in a rotating drum. To help the physical understanding of the flow structure in a rotating drum, on the other hand, the velocity fields (*u*, *v*) measured in the cartesian coordinates (*x*, *y*) are converted into those in the polar coordinates (*r*, ) as $\:{u}_{r}\left(r,\theta\:\right)=u\text{cos}\theta\:+v\text{sin}\theta\:$ and $\:{u}_{\theta\:}\left(r,\theta\:\right)=-u\text{sin}\theta\:+v\text{cos}\theta\:$ (Fig. 11) for further discussion.

While measuring the velocity distribution using a particle image velocimetry, there are several sources to cause the experimental uncertainties^29^. For example, the magnification factor (*M*) associated with the optical configuration, control of the time interval ($\:\varDelta\:t$) between successive images, and the uncertainty of measuring the particle displacement ($\:\varDelta\:s$) during $\:\varDelta\:t$ would contribute to the overall uncertainty of PIV measurements. Based on the error propagation theory, this can be expressed as $\:{({\delta\:\left(M\right)}^{2}+{\delta\:(\varDelta\:t)}^{2}+{\delta\:(\varDelta\:s)}^{2})}^{0.5}$, where δ(*x*) denotes the percentage errors in obtaining the variable (*x*)^30,31^. During the calibration using a two-dimensional calibration target, it was calculated that δ(M) is approximately 3.12–3.44%, with *M* = 322.6–357.1 μm/pixel. For the present time separation of $\:\varDelta\:t$ = 2.5 msec, the corresponding δ($\:\varDelta\:t$) was measured to be negligibly small. Finally, δ($\:\varDelta\:s$) associated with the spatial resolution of 275 pixels was estimated to be approximately 3.03% for $\:\varDelta\:s$ = 266.67 pixels, which corresponds to representative velocity 0.11 m/s at the free surface. In overall, the uncertainty of the present particle image velocimetry measurements is evaluated to be approximately 4.35–4.59%.

**Fig. 13:**
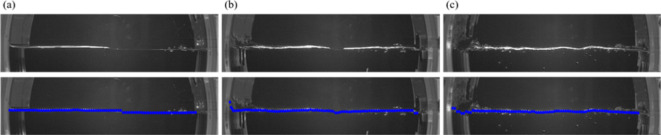
Raw images of the air-water interface (top) and post-processed results (bottom) to measure change in the free surface (bottom) at (**a**) *N* = 10 rpm; (**b**) 30 rpm; (**c**) 50 rpm. In the bottom images, the blue color denotes the instantaneous position of the detected free surface.

### Visualization and detection of the free surface

To visualize and detect the instantaneous position of the free surface, we utilized brightness variations measured at the free surface in the images. A high-speed camera with a 50-mm lens (SAMYANG) was used and a plane white-colored LED (50 W) and a halogen lamp were employed as light sources to illuminate the free surface (see Fig. S14 in the supplementary materials). The images were acquired at a rate of 400 frames per second with a spatial resolution of 960 $\:\times\:$ 960 pixels, and more than 14,000 images were taken and analyzed to achieve the statistically converged data and minimize measurement error. As shown in Fig. 13, the free surface appears significantly brighter than other regions due to the reflection at the air-water interface, allowing for a clear detection of free surface in the images. After removing potential noise from the image by subtracting the background from the raw image and applying an image-sharpening algorithm, we applied the Otsu method^32^ to obtain a binarized image. Based on the variation in binarized pixel intensity, $\:{I}_{b}\left(x,y,t\right)$, the maximum $\:y$-value $\:{y}_{M}(x,t)$, which satisfies $\:{I}_{b}\left(x,y,t\right)=1.0$ for each $\:x$ in the processed image, is detected. The change in the height of free surface is defined as $\:{y}_{f}\left(x,t\right)={y}_{M}\left(x,t\right)-{y}_{o}$, where $\:{y}_{o}$ represents the stationary position.

## Electronic supplementary material

Below is the link to the electronic supplementary material.

Supplementary Material 1

## Data Availability

All data generated or analyzed during this study are included in this published article and supplementary information files.
