# Supplementary material for: Liquid flows induced in a rotating drum with different fill ratios
Source: Sci Rep. 2025 Jan 14;15:1959. doi: 10.1038/s41598-024-84579-7 (PMC11733300; doi:10.1038/s41598-024-84579-7)
Supplement: Supplementary file 1 — Supplementary Material 1 [file 41598_2024_84579_MOESM1_ESM.pdf]

**Supplementary Materials for**

# Liquid flows induced in a rotating drum with different fill ratios

Daeun Lee[1,†], Jaebeen Lee[1,†], Seok Min Choi[2], Sangtak Lee[2], and Hyungmin Park[1,3,*]

[1]Department of Mechanical Engineering, Seoul National University, Seoul 08826, Korea

[2]Samsung Research, Samsung Electronics Co., Ltd., Seoul 06765, Korea

[3]Institute of Advanced Machines and Design, Seoul National University, Seoul 08826, Korea

[†]Equally contributed to this work.

[*]Author to whom correspondence should be addressed: hminpark@snu.ac.kr

## Figure list

Fig. S1. Radial profiles of the time-averaged azimuthal velocity, $\bar{u}_\theta$ (black circle), and radial velocity, $\bar{u}_r$ (white triangle), measured at *z/W* = 0: (a) *N* = 10 rpm; (b) 30 rpm; (c) 50 rpm.

Fig. S2. Contours of (a-c) the time-averaged azimuthal velocity and (d-f) turbulent kinetic energy ($TKE = 0.5(u_r'^2 + u_\theta'^2)$) for a fully-filled case measured at the plane of z/*W* = 0.25: (a, d) *N* = 10 rpm; (b, e) 30 rpm; (c, f) 50 rpm.

Fig. S3. Contours of time-averaged azimuthal velocity for different water height (*H*) and rotation speed (*N*) at *z*/*W* = 0: (a-c) *H*/*D* = 0.25; (d-f) 0.375; (g-i) 0.5. (a, d, g) *N* = 10 rpm; (b, e, h) 30 rpm; (c, f, i) 50 rpm.

Fig. S4. Contours of time-averaged radial velocity for different water height (*H*) and rotation speed (*N*) at *z*/*W* = 0: (a-c) *H*/*D* = 0.25; (d-f) 0.375; (g-i) 0.5. (a, d, g) *N* = 10 rpm; (b, e, h) 30 rpm; (c, f, i) 50 rpm.

Fig. S5. Contours of axial vorticity for different water height (*H*) and rotation speed (*N*) at *z/W* = 0: (a-c) *H/D* = 0.25; (d-f) 0.375; (g-i) 0.5; (a, d, g) *N* = 10 rpm; (b, e, h) 30 rpm; (c, f, i) 50 rpm.

Fig. S6. Contours of the root-mean-square fluctuating azimuthal ($u_\theta'$) velocity for different water height (*H*) and rotation speed (*N*) at *z*/*W* = 0: (a-c) *H*/*D* = 0.25; (d-f) 0.375; (g-i) 0.5. (a, d, g) *N* = 10 rpm; (b, e, h) 30 rpm; (c, f, i) 50 rpm.

Fig. S7. Contours of the root-mean-square fluctuating radial ($u_r'$) velocity for different water height (*H*) and rotation speed (*N*) at *z*/*W* = 0: (a-c) *H*/*D* = 0.25; (d-f) 0.375; (g-i) 0.5. (a, d, g) *N* = 10 rpm; (b, e, h) 30 rpm; (c, f, i) 50 rpm.

Fig. S8. Contours of the normalized Reynolds stress for different water height (*H*) and rotation speed (*N*) at *z*/*W* = 0: (a-c) *H*/*D* = 0.25; (d-f) 0.375; (g-i) 0.5. (a, d, g) *N* = 10 rpm; (b, e, h) 30 rpm; (c, f, i) 50 rpm.

Fig. S9. Contours of the normalized turbulent kinetic energy for different water height (*H*) and rotation speed (*N*) at *z*/*W* = 0: (a-c) *H*/*D* = 0.25; (d-f) 0.375; (g-i) 0.5. (a, d, g) *N* = 10 rpm; (b, e, h) 30 rpm; (c, f, i) 50 rpm.

Fig. S10. Radial profiles of the time-averaged azimuthal velocity, $\bar{u}_\theta(r)$ in Cartesian coordinates, at specific angles ($\theta$), measured at *z/W* = 0.25 (a, c) and 0 (b, d): (a, b) *H/D* = 0.25 and *N* = 10 rpm; (c, d) *H/D* = 0.5 and *N* = 50 rpm.

Fig. S11. Azimuthal profiles of the time-averaged radial velocity, $\bar{u}_r(\theta)$ in Cartesian coordinates, at specific radial positions ($r$), measured at *z*/*W* = 0.25 (a, c) and 0 (b, d): (a, b) *H*/*D* = 0.25 and *N* = 10 rpm; (c, d) *H*/*D* = 0.5 and *N* = 50 rpm.

Fig. S12. Schematic diagram for the experimental setup for the rotating drum in side (y-z) planes.

Fig. S13. Development of the instantaneous velocity field for a fully-filled rotating drum at *N* = 10 rpm after (a) 10 seconds; (b) 1 minute; (c) 2 minutes; (d) 3 minutes; (e) 4 minutes; (f) 10 minutes.

Fig. S14. Schematic diagram for the experimental setup to visualize changes in the position of the free surface in the rotating flow.

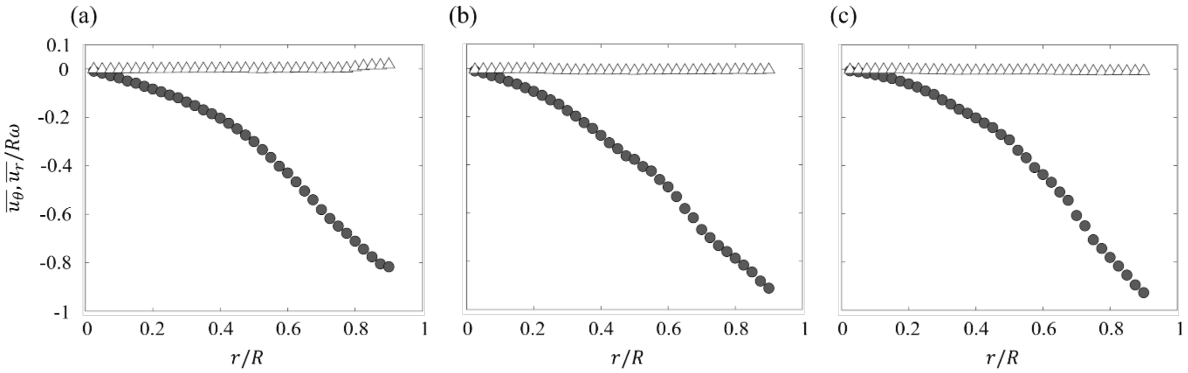


Fig. S1. Radial profiles of the time-averaged azimuthal velocity, $\bar{u}_\theta$ (black circle), and radial velocity, $\bar{u}_r$ (white triangle), measured at $z/W = 0$: (a) $N = 10$ rpm; (b) 30 rpm; (c) 50 rpm.

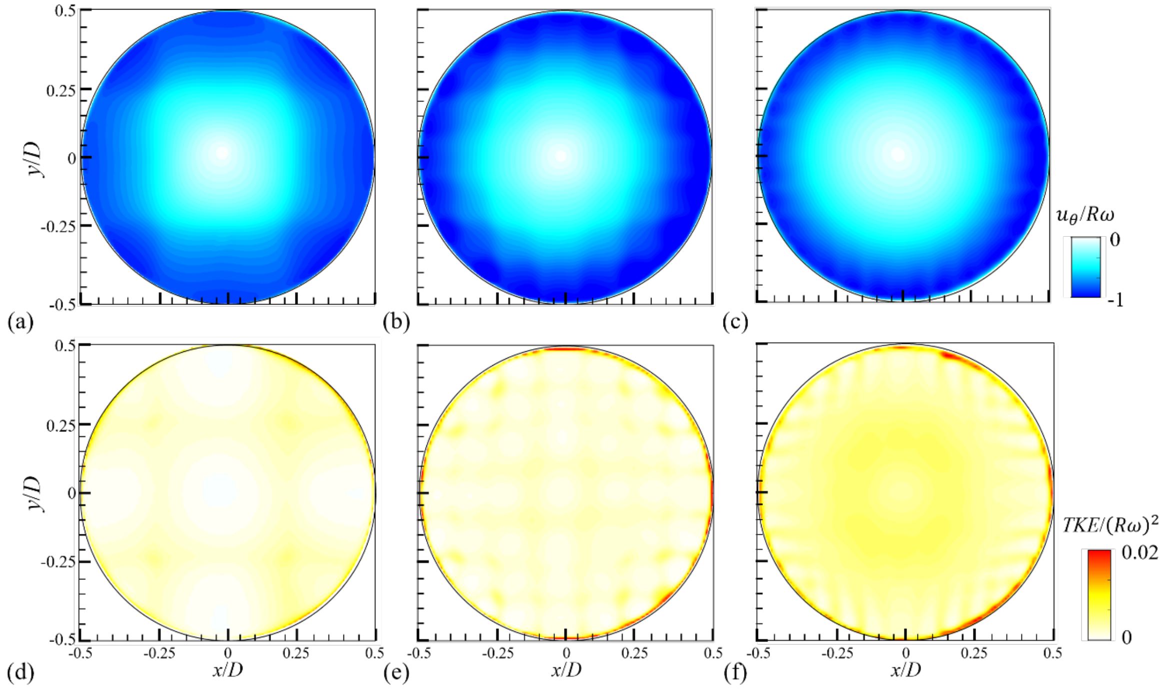


Fig. S2. Contours of (a-c) the time-averaged azimuthal velocity and (d-f) turbulent kinetic energy ($TKE$ = $\mathbf{0.5(u_r'^2 + u_\theta'^2)}$) for a fully-filled case measured at the plane of $z/W = 0.25$: (a, d) $N = 10$ rpm; (b, e) 30 rpm; (c, f) 50 rpm.

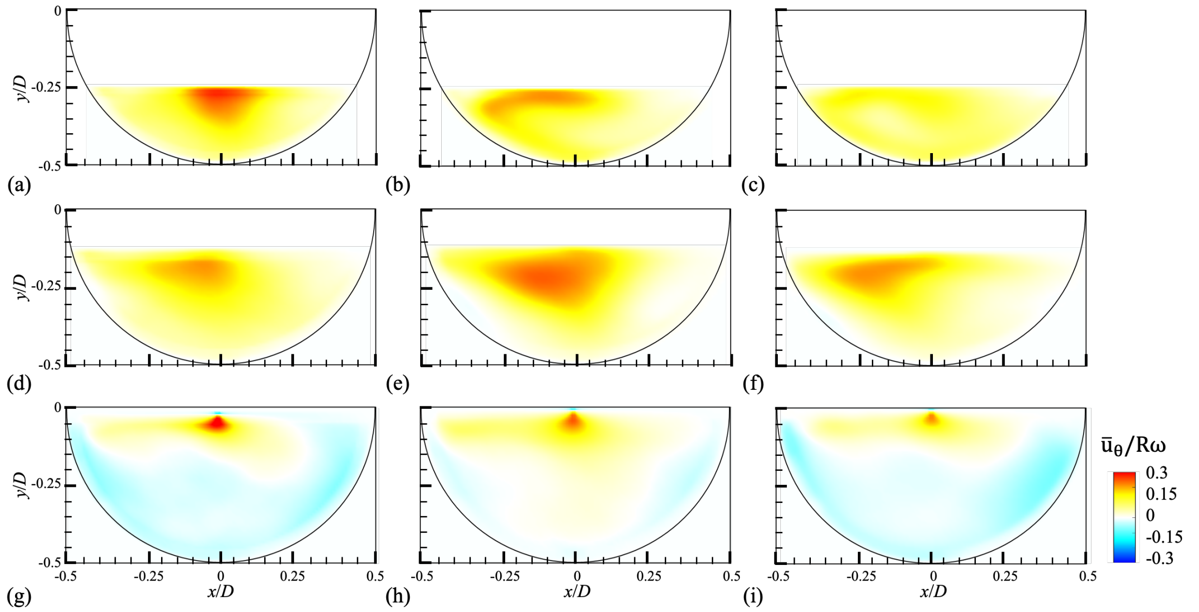


Fig. S3. Contours of time-averaged azimuthal velocity for different water height (*H*) and rotation speed (*N*) at $z/W = 0$: (a-c) $H/D = 0.25$; (d-f) 0.375; (g-i) 0.5. (a, d, g) $N = 10$ rpm; (b, e, h) 30 rpm; (c, f, i) 50 rpm.

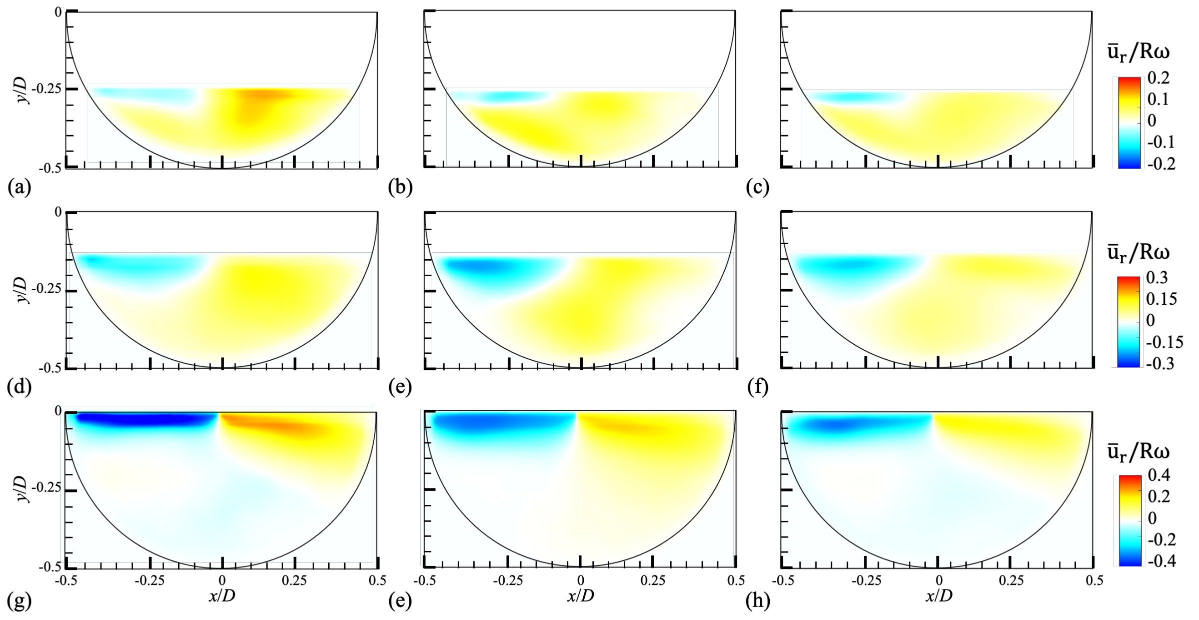


Fig. S4. Contours of time-averaged radial velocity for different water height (*H*) and rotation speed (*N*) at $z/W = 0$: (a-c) $H/D = 0.25$; (d-f) 0.375; (g-i) 0.5. (a, d, g) $N = 10$ rpm; (b, e, h) 30 rpm; (c, f, i) 50 rpm.

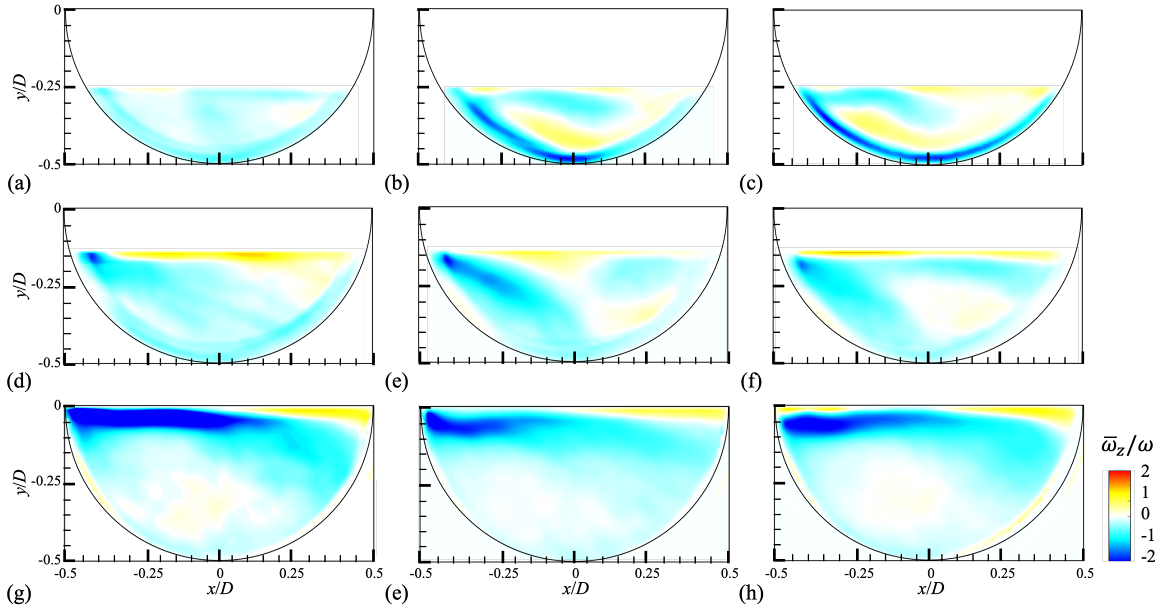


Fig. S5. Contours of axial vorticity for different water height ($H$) and rotation speed ($N$) at $z/W = 0$: (a-c) $H/D = 0.25$; (d-f) 0.375; (g-i) 0.5; (a, d, g) $N = 10$ rpm; (b, e, h) 30 rpm; (c, f, i) 50 rpm.

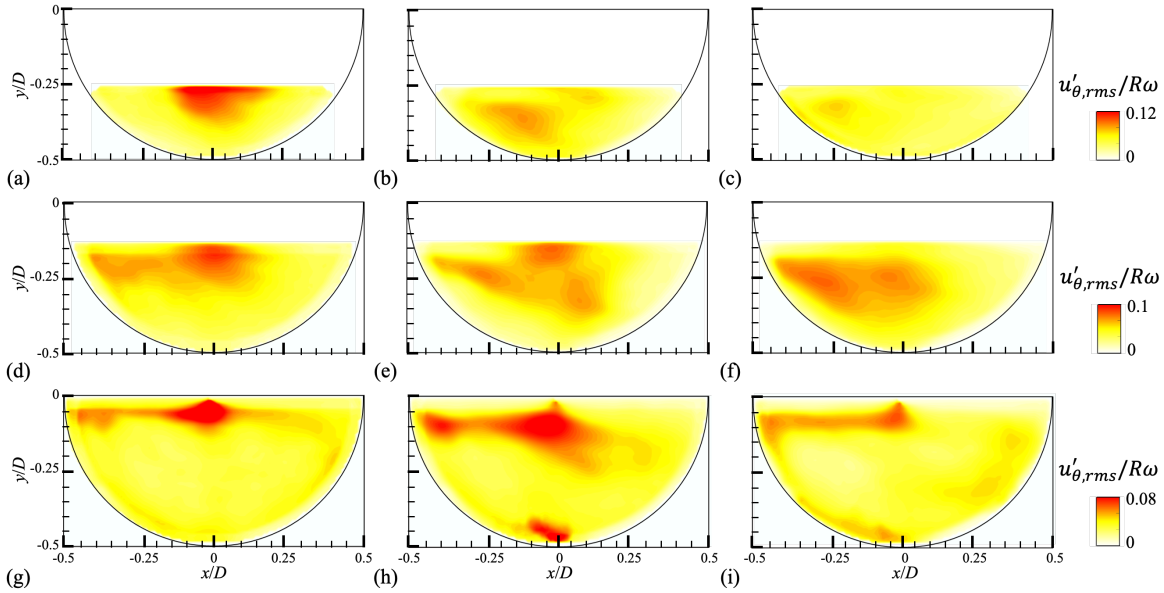


Fig. S6. Contours of the root-mean-square fluctuating azimuthal ($u'_\theta$) velocity for different water height ($H$) and rotation speed ($N$) at $z/W = 0$: (a-c) $H/D = 0.25$; (d-f) 0.375; (g-i) 0.5. (a, d, g) $N = 10$ rpm; (b, e, h) 30 rpm; (c, f, i) 50 rpm.

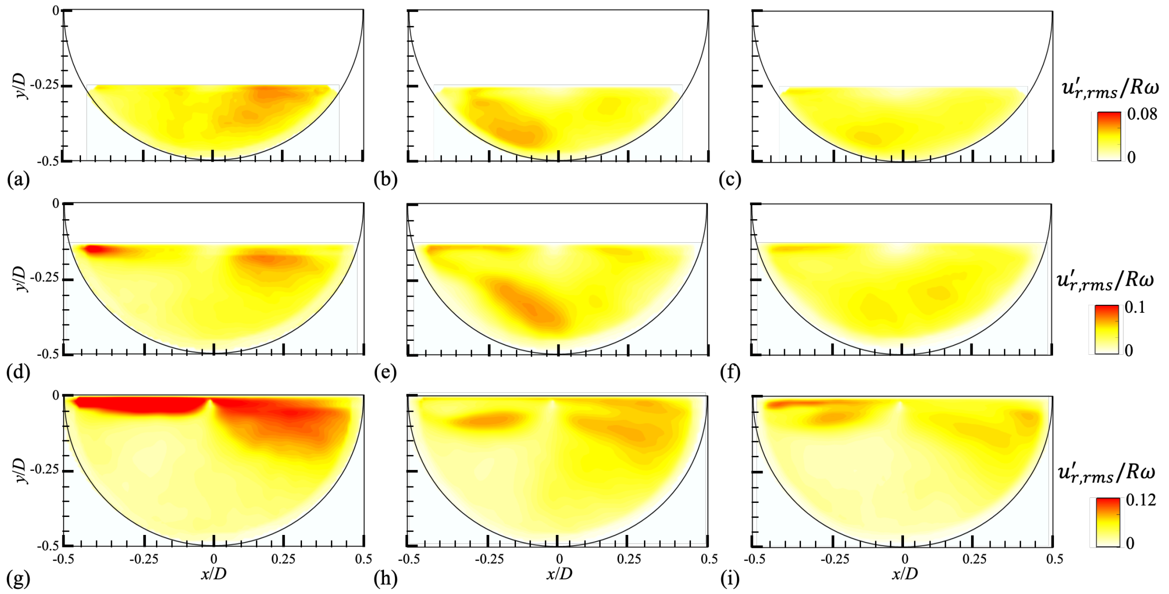


Fig. S7. Contours of the root-mean-square fluctuating radial ($u'_r$) velocity for different water height ($H$) and rotation speed ($N$) at $z/W = 0$: (a-c) $H/D = 0.25$; (d-f) 0.375; (g-i) 0.5. (a, d, g) $N = 10$ rpm; (b, e, h) 30 rpm; (c, f, i) 50 rpm.

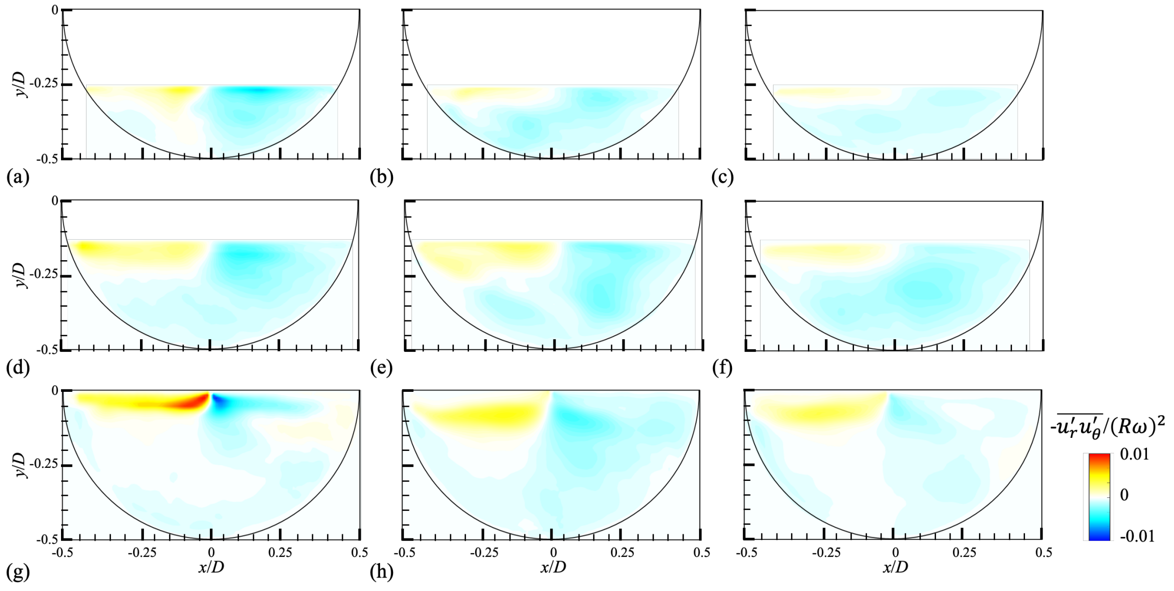


Fig. S8. Contours of the normalized Reynolds stress for different water height ($H$) and rotation speed ($N$) at $z/W = 0$: (a-c) $H/D = 0.25$; (d-f) 0.375; (g-i) 0.5. (a, d, g) $N = 10$ rpm; (b, e, h) 30 rpm; (c, f, i) 50 rpm.

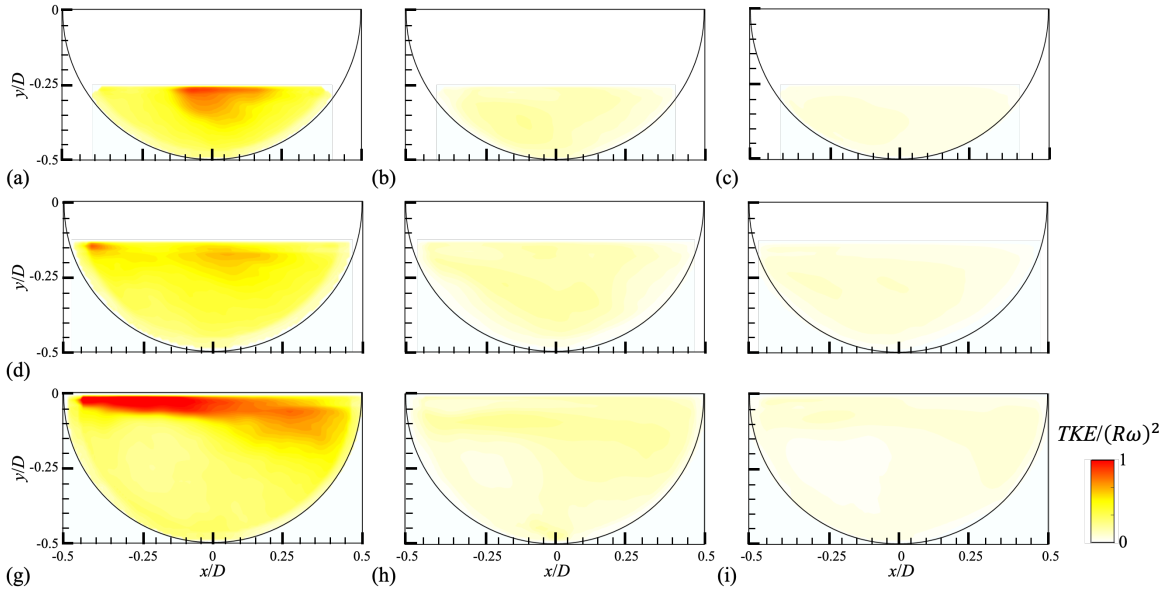


Fig. S9. Contours of the normalized turbulent kinetic energy for different water height (*H*) and rotation speed (*N*) at *z*/*W* = 0: (a-c) *H*/*D* = 0.25; (d-f) 0.375; (g-i) 0.5. (a, d, g) *N* = 10 rpm; (b, e, h) 30 rpm; (c, f, i) 50 rpm.

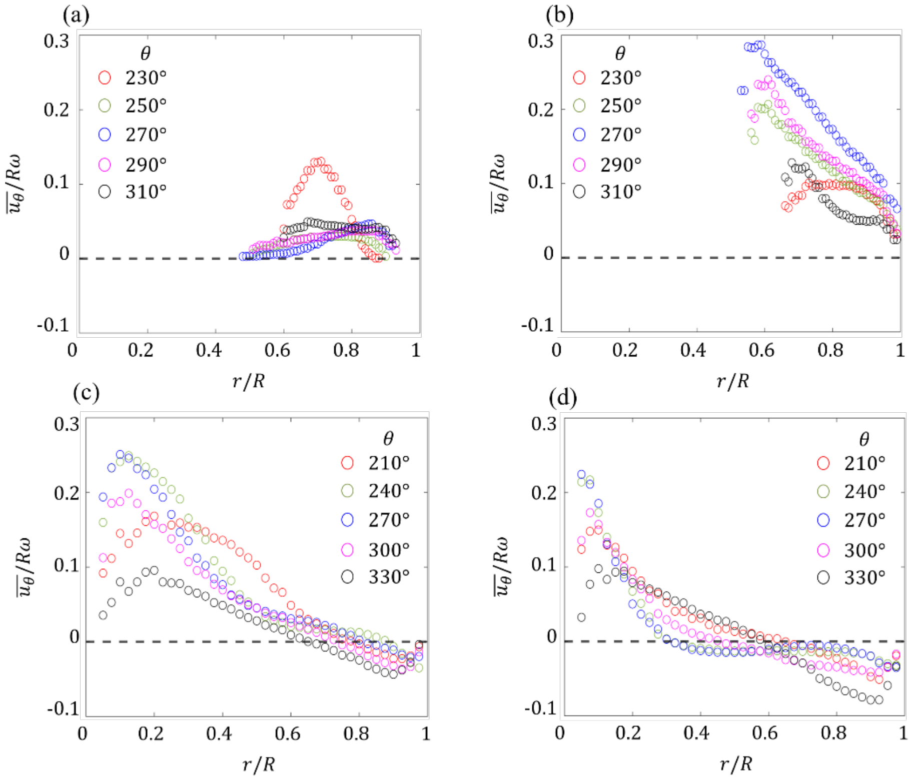


Fig. S10. Radial profiles of the time-averaged azimuthal velocity, $\bar{u}_\theta(r)$ in Cartesian coordinates, at specific angles ($\theta$), measured at *z*/*W* = 0.25 (a, c) and 0 (b, d): (a, b) *H*/*D* = 0.25 and *N* = 10 rpm; (c, d) *H*/*D* = 0.5 and *N* = 50 rpm.

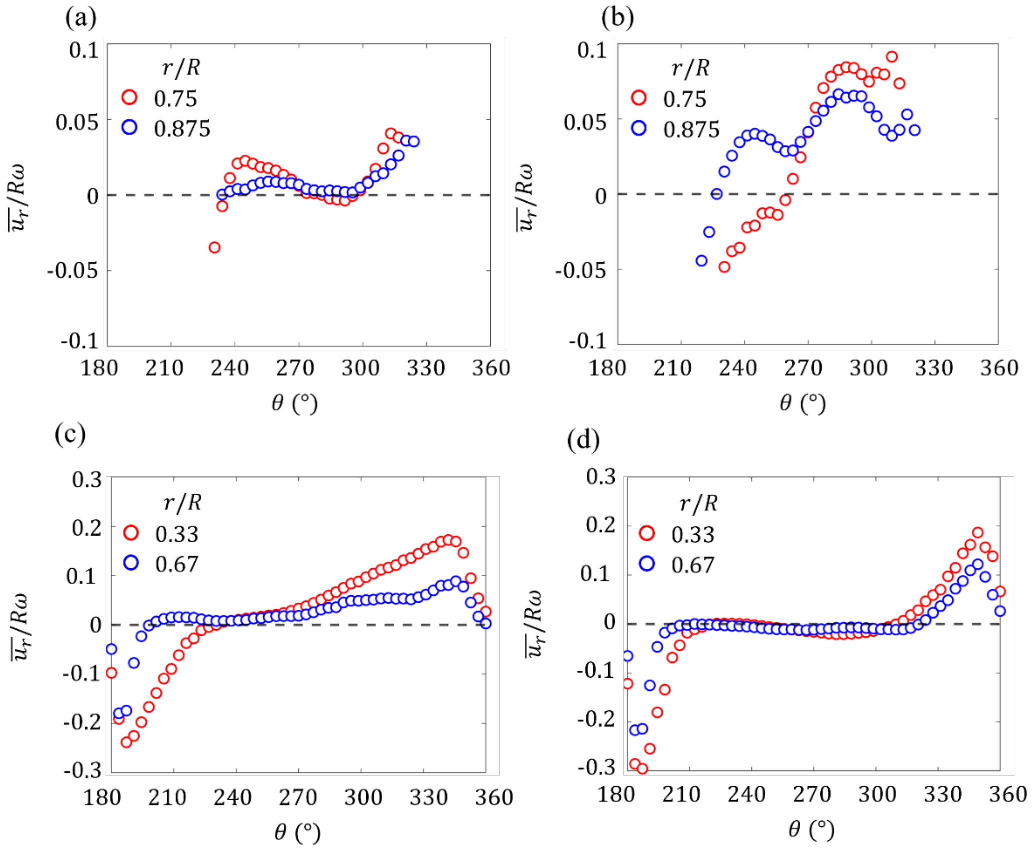


Fig. S11. Azimuthal profiles of the time-averaged radial velocity, $\bar{u}_r(\theta)$ in Cartesian coordinates, at specific radial positions ($r$), measured at $z/W$ = 0.25 (a, c) and 0 (b, d): (a, b) $H/D$ = 0.25 and $N$ = 10 rpm; (c, d) $H/D$ = 0.5 and $N$ = 50 rpm.

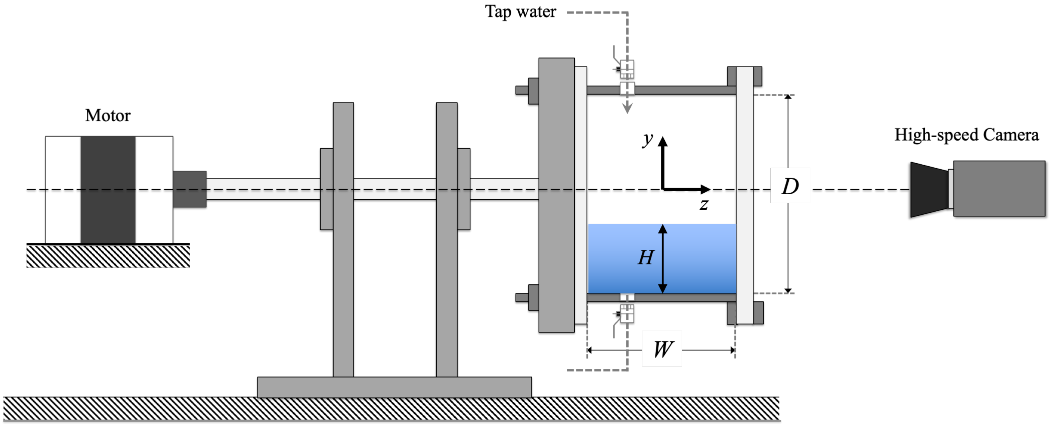


Fig. S12. Schematic diagram for the experimental setup for the rotating drum in side (y-z) planes.

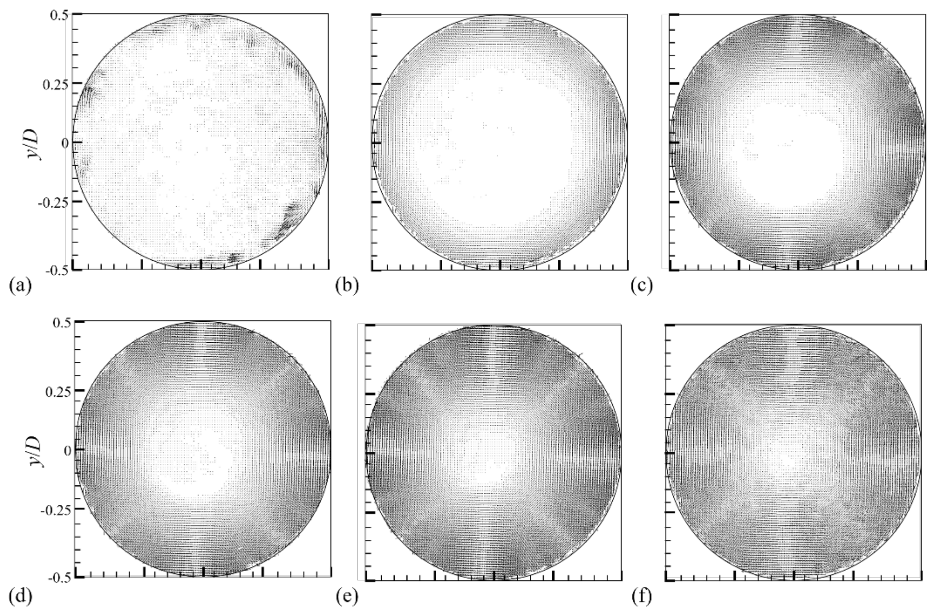


Fig. S13. Development of the instantaneous velocity field for a fully-filled rotating drum at $N = 10$ rpm after (a) 10 seconds; (b) 1 minute; (c) 2 minutes; (d) 3 minutes; (e) 4 minutes; (f) 10 minutes.

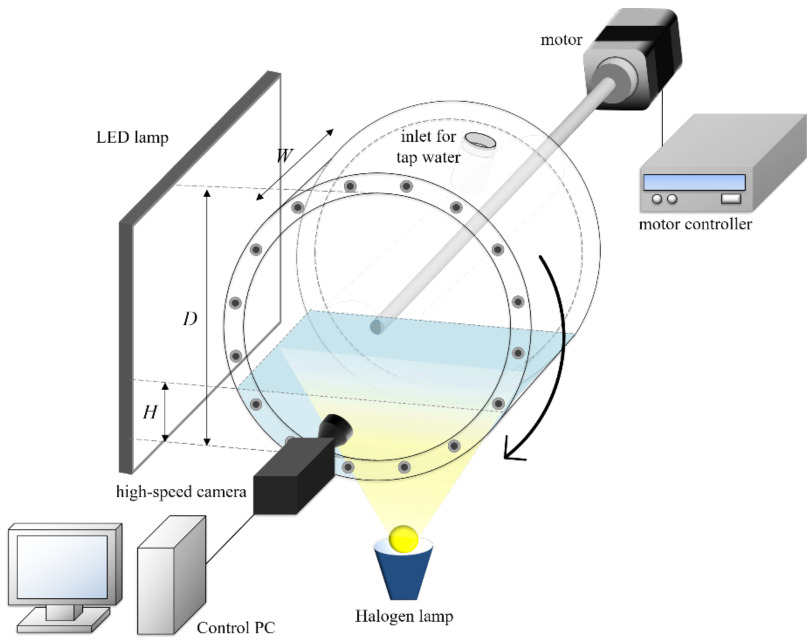


Fig. S14. Schematic diagram for the experimental setup to visualize changes in the position of the free surface in the rotating flow.

**References**


1. Johnson, R. E. Steady-state coating flows inside a rotating horizontal cylinder. J. Fluid Mech. 190, 321-342 (1988).
2. Denis, C., Hemati, M., Chulia, D., Lanne, J. Y., Buisson, B., Daste, G. & Elbaz, F. A model of surface renewal with application to the coating of pharmaceutical tablets in rotary drums. Powder Tech. 130, 174-180 (2003).
3. Cameron, I. T., Wang, F. Y., Immanuel, C. D. & Stepanek, F. Process systems modelling and applications in granulation: A review. Chem. Eng. Sci. 60, 3723-3750 (2005).
4. Wu, W., Chen, K. & Tsotsas, E. Prediction of particle mixing in rotary drums by a DEM data-driven PSO-SVR model. Powder Tech. 434, 119365 (2024).
5. Mellmann, J., Specht, E. & Liu, X. Prediction of rolling bed motion in rotating cylinders. AIChE J. 50, 2783-2793 (2004).
6. Liu, X. Y., Specht, E., Gonzalez, O. G. & Walzel, P. Analytical solution for the rolling-mode granular motion in rotary kilns. Chem. Eng. Process.: Process Intensif. 45, 515-521 (2006).
7. Papapetrou, T. N., Bieberle, M., Barthel, F., Hampel, U. & Lecrivain, G. Investigating binary granular mixing in a rotating drum using ultrafast X-ray computed tomography. Powder Tech. 119964 (2024).
8. Henein, H., Brimacombe, J. K. & Watkinson, A. P. Experimental study of transverse bed motion in rotary kilns. Metall. Mater. Trans. B 14, 191-205 (1983).
9. Yang, R. Y., Yu, A. B., McElroy, L. & Bao, J. Numerical simulation of particle dynamics in different flow regimes in a rotating drum. Powder Tech. 188, 170-177 (2008).
10. Deiber, J. A. & Cerro, R. L. Viscous flow with a free surface inside a horizontal rotating drum. I. Hydrodynamics. Ind. Eng. Chem. Res. 15, 102-110 (1976).
11. Jiang, Z., Wang, G., Ge, S. & Yuan, H. Numerical simulation of the dynamical behavior of a spinning cylinder partially filled with liquid. Phys. Fluids 35, 117112 (2023).
12. Ivanova, A. A., Kozlov, V. G. & Chigrakov, A. V. Dynamics of a fluid in a rotating horizontal cylinder. Fluid Dyn. 39, 594-604 (2004).
13. Olitskii, A. F., Shrager, G. R. & Yakutenok, V. A. Viscous flow in a partially-filled horizontal cylinder rotating at constant speed. Fluid Dyn. 28, 315-319 (1993).
14. Thoroddsen, S. T. & Mahadevan, L. Experimental study of coating flows in a partially-filled horizontally rotating cylinder. Exp Fluids 23, 1-13 (1997).

15. Murakami, M., Kikuyama, K. & Nishibori, K. Flow in axially rotating pipes. Nagoya University, Faculty of Engineering, Memoirs 35, 1-35 (1983).
16. Haji-Sheikh, A., Lakshimanarayanan, R., Lou, D. Y. & Ryan, P. J. Confined flow in a partially-filled rotating horizontal cylinder. J. Fluids Eng. 106, 270-278 (1984).
17. Böhme, G., Pokriefke, G. & Müller, A. Viscous flow phenomena in a partially filled rotor-stator system. Arch. Appl. Mech. 75, 619-634 (2006).
18. Dyakova, V. & Polezhaev, D. Oscillatory and steady flows in the annular fluid layer inside a rotating cylinder. Shock Vib. 2016, 162368 (2016).
19. Greenspan, H. P. The theory of rotating fluids. Cambridge University Press (1969).
20. Watkins, W. B., & Hussey, R. G. Spin-up from rest in a cylinder. Phys. Fluids, 20, 1596-1604 (1977)
21. Phillips, O. M. Centrifugal waves. J. Fluid Mech., 7, 340-352 (1960).
22. Orr, F. M., & Scriven, L. E. Rimming flow: numerical simulation of steady, viscous, free-surface flow with surface tension. J. Fluid Mech., 84, 145-165 (1978).
23. Romanò, F., Hajisharifi, A. & Kuhlmann, H. C. Cellular flow in a partially filled rotating drum: regular and chaotic advection. J. Fluid Mech. 825, 631-650 (2017).
24. Gilpin, W., Prakash, V. N. & Prakash, M. Flowtrace: simple visualization of coherent structures in biological fluid flows. J. Exp. Biol. 220, 3411-3418 (2017).
25. Kim, N., Park, H. & Do, H. Evolution of cavitation bubble in tap water by continuous-wave laser focused on a metallic surface. Langmuir 35, 3308-3318 (2019).
26. Arntz, M. M. H. D., den Otter, W. K., Briels, W. J., Bussmann, P. J. T., Beeftink, H. H. & Boom, R. M. Granular mixing and segregation in a horizontal rotating drum: a simulation study on the impact of rotational speed and fill level. AIChE J. 54, 3133-3146 (2008).
27. Scarano, F. Iterative image deformation methods in PIV. Meas Sci Technol. 13, R1 (2001).
28. Westerweel, J. & Scarano, F. Universal outlier detection for PIV data. Exp. Fluids 39, 1096-1100 (2005).
29. Raffel, M., Willert, C. E. Kompenhans, J. 2007 Particle Image Velocimetry: A Practical Guide. Springer.
30. Choi, D. & Park, H. Flow-structure interaction of a starting jet through a flexible circular nozzle. J. Fluid Mech. 949, A39 (2022).
31. Yeo, J. & Park, H. Bubble-bubble interactions in two-dimensional bubble-chain flows. Int. J. Multiphas. Flow 171, 104665 (2024).

32. Otsu, N. A threshold selection method from gray-level histograms. IEEE Trans. Syst. Man. Cybern. 9, 62-66 (1979).
